# Nuclear genome-wide associations with mitochondrial heteroplasmy

**DOI:** 10.1126/sciadv.abe7520

**Published:** 2021-03-17

**Authors:** Priyanka Nandakumar, Chao Tian, Jared O’Connell, 23andMe Research Team, David Hinds, Andrew D. Paterson, Neal Sondheimer

**Affiliations:** 1 23andMe Inc., 223 N Mathilda Ave, Sunnyvale, CA, USA.; 2 Program in Genetics and Genome Biology, The Hospital for Sick Children, Toronto, ON, Canada.; 3 Divisions of Epidemiology and Biostatistics, Dalla Lana School of Public Health, University of Toronto, Toronto, ON, Canada.; 4 Departments of Paediatrics and Molecular Genetics, University of Toronto, Toronto, ON, Canada.

## Abstract

The role of the nuclear genome in maintaining the stability of the mitochondrial genome (mtDNA) is incompletely known. mtDNA sequence variants can exist in a state of heteroplasmy, which denotes the coexistence of organellar genomes with different sequences. Heteroplasmic variants that impair mitochondrial capacity cause disease, and the state of heteroplasmy itself is deleterious. However, mitochondrial heteroplasmy may provide an intermediate state in the emergence of novel mitochondrial haplogroups. We used genome-wide genotyping data from 982,072 European ancestry individuals to evaluate variation in mitochondrial heteroplasmy and to identify the regions of the nuclear genome that affect it. Age, sex, and mitochondrial haplogroup were associated with the extent of heteroplasmy. GWAS identified 20 loci for heteroplasmy that exceeded genome-wide significance. This included a region overlapping mitochondrial transcription factor A (*TFAM*), which has multiple roles in mtDNA packaging, replication, and transcription. These results show that mitochondrial heteroplasmy has a heritable nuclear component.

## Teaser

This study identifies regions of the nuclear genome that affect heteroplasmy, the mixture of different mitochondrial DNA.

## INTRODUCTION

Human mitochondrial DNA (mtDNA) is the maternally inherited genome that is dedicated to the generation of cellular energy through oxidative phosphorylation (*1*). mtDNA is small, expressing only 13 protein-coding genes, along with the ribosomal and transfer RNAs required for their translation. Despite its size, sequence changes in mtDNA and their interaction with the nuclear genome may have outsized impact upon health and disease.

Control of the mtDNA copy number per cell is variable between different cell types, with some human cells containing no mtDNA and others containing thousands of copies. As a multicopy genome, mutation of mtDNA leads to a state of mitochondrial heteroplasmy (MtHz) where mtDNA with distinct sequences coexist. Once viewed as uncommon, it has been recognized that MtHz is widely present in humans (*2*). MtHz can be transmitted through the maternal germ line so that mother and offspring are heteroplasmic at the same position(s) (*3*). In addition, somatic MtHz occurs in the context of aging and tissue damage (*4*, *5*).

Most of the pathogenic variants in mtDNA are heteroplasmic. Homoplasmic mutations are less common and cause disorders such as Leber’s hereditary optic neuropathy, which does not impair reproductive fitness. For heteroplasmic disease-causing variants, the ratio of the pathogenic variant to the total mitochondrial pool (commonly referred to as load) plays an important role in the penetrance and expressivity of the disorders (*6*). Below a variant-specific threshold, symptoms are not observed. Above the therapeutic threshold, symptoms become progressively more severe. Therapeutic shifting of MtHz toward the wild-type allele has long been proposed (*7*), and several approaches are under investigation (*8*–*10*), but there are no clinically approved means to directionally alter MtHz to treat disease. In addition, there is evidence from both quantitative models and animal studies that MtHz itself is deleterious and that homoplasmic states are preferred, even when the two mtDNA sequences present contain no pathogenic variants (*11*, *12*).

Conversely, MtHz may serve a beneficial role by allowing a transitional state between two mitochondrial genotypes. As a uniparentally inherited genome, new alleles and new combinations of alleles are introduced via mutation to a heteroplasmic state. Commonly inherited sets of mitochondrial polymorphisms, known as haplogroups, have arisen during the course of human migration and evolution, likely in response to challenges encountered in new environments (*13*). Mitochondrial polymorphisms associated with mitochondrial haplogroups are known to affect mtDNA copy number, rates of mitochondrial transcription, and capacity for oxidative phosphorylation (*14*–*16*).

Despite the importance of MtHz in health and disease, its origins and the balance between its benefits and consequences are incompletely understood. We sought to further our understanding of MtHz by evaluating nuclear loci that affect MtHz in a large sample.

## RESULTS

### Dataset and analytical strategy

**Fig. 1:**
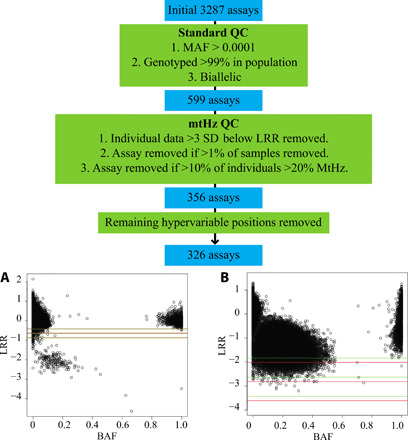
Evaluation mitochondrial SNPs on the 23andMe v4 array. The array includes 3287 positions across the mitochondrial genome. These were pruned to keep biallelic variants, with >99% call rate across the test population and minor allele frequency (MAF) > 0.001. Calls from individual participants at single SNPs were removed, where the intensity (LRR, log_2_ R ratio) was >3 SDs below the mean intensity for all individuals at that position. Where a position had >1% of samples fail these criteria, the position was removed for all individuals. (**A**) Well-performing assay with heteroplasmic samples between two homoplasmic clusters and a small number of samples with intensity >3 SDs below the mean value (lowest line) where individual calls were excluded. SD lines are shown for both the (A) (green) and (B) (red) allele assay. Assays that identified excessive heteroplasmy with poor homoplasmic clusters were entirely removed. (**B**) Poorly performing assay where >10% of the individuals in the dataset had MtHz of >20% [B-allele frequency (BAF) values, 0.2 to 0.8]. For this position, data from all individuals were removed from the analysis.

We used genome-wide genotyping data from saliva samples of 982,072 individuals of European ancestry who were participants in the research program of 23andMe (Table 1), a personal genomics and biotechnology company. MtDNA is densely genotyped with 3287 single-nucleotide polymorphisms (SNPs) assayed. Quality control (QC) measures were applied to remove assays that genotyped poorly due to either a lack of hybridization or inaccurate discrimination of the alleles. Ultimately, 326 mtDNA SNPs were evaluated (Fig. 1). MtHz values were calculated as the ratio of the lesser allele intensity to the total intensity at that position so that the maximum possible heteroplasmy value was 0.5. We used mother-offspring duos to examine pairs of points where the mother had a heteroplasmy value of >5% (fig. S1; *n* = 28,963 pairs). There was a correlation in maternal-offspring values as would be expected for an inherited heteroplasmy [Spearman’s *r* = 0.1846, *P* = 3.4 × 10^−70^ where maternal B-allele frequency (BAF) value was <0.5, and Spearman’s *r* = 0.1800, *P* = 8.9 × 10^−185^ where the maternal BAF was >0.5].

**Table 1:** Characteristics of the subject sex and age groups by mean MtHz quartile.

**Mean MtHz**	**Total**	**Male**	**Female**	**Age <30**	**Age 30–45**	**Age 45–60**	**Age >60**
<0.0046	245,518	113,906	131,612	23,157	58,126	64,360	99,875
0.0046–0.0074	245,518	114,262	131,256	23,408	58,086	65,009	99,015
0.0074–0.012	245,518	116,018	129,500	23,983	59,018	65,330	97,187
>0.012	245,518	118,107	127,411	24,292	58,685	64,888	97,653

**Fig. 2:**
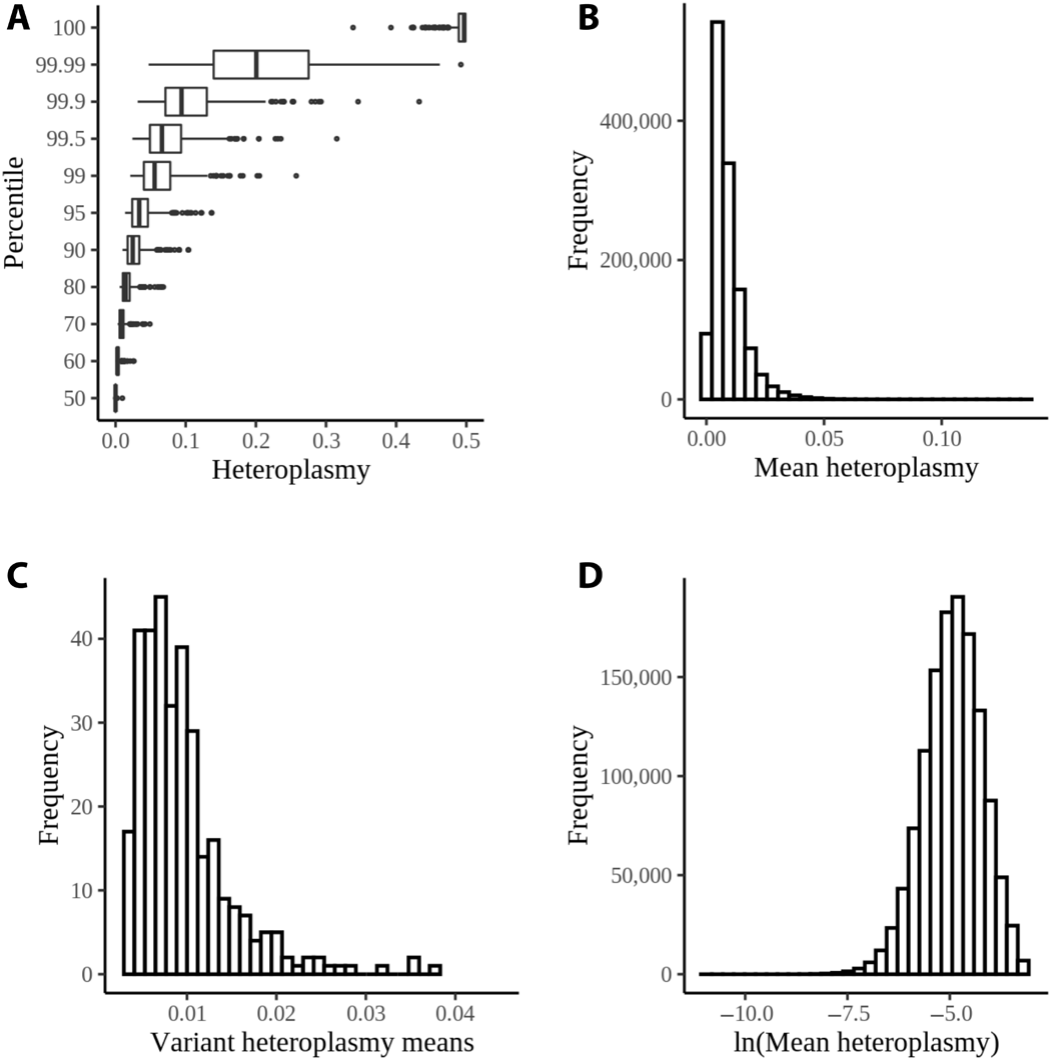
Quantitation of MtHz data. (**A**) MtHz plotted by percentile within the population for each of the mitochondrial positions evaluated. Highly heteroplasmic individuals were observed at every position. (**B**) Mean mitochondrial heteroplasmy, averaged across the evaluated positions, for individuals in the dataset. (**C**) Distribution of mean MtHz values across the 326 tested mitochondrial positions in the QC subset. (**D**) Natural log transformation of mean MtHz with outlier removal at 99.5%.

MtHz was widely present, and all assayed positions had individuals with >25% MtHz (Fig. 2A). Because our approach did not evaluate all positions in the mitochondrial genome, we quantified MtHz for each individual as the mean value across all 326 positions assayed. The mean MtHz for all individuals evaluated was 0.00744 (interquartile range = 0.0046 to 0.012; Fig. 2B). Notably, the mean heteroplasmy values were not driven by a subset of the mitochondrial positions, as heteroplasmy averaged across individuals at each position was tightly distributed (Fig. 2C).

### Impact of age, sex, and mitochondrial haplogroup on MtHz

To evaluate the impact of genetic and nongenetic factors upon mean MtHz, we calculated the natural log-transformed mean MtHz value winsorized at 99.5% to remove the influence of outliers (Fig. 2D). An initial model was generated to evaluate influences upon mean MtHz (Table 2). The variance of allelic intensity for homozygous autosomal SNPs (“autosomal variance”) was strongly positively associated with MtHz. This was anticipated as samples with high autosomal variance at homozygous positions likely had technical characteristics that indicate noisier samples. This noise overestimates MtHz, and the use of autosomal variance in the genome-wide association study (GWAS) corrects for minor differences in sample quality.

**Table 2:** Effect of covariates on mean MtHz. The pc values are from principal components analysis. Autosomal variance refers to the variability from homozygosity at autosomal positions. Haplogroup values are compared to haplogroup H (*n* = 72,363). The number of individuals with each haplogroup is noted.

**Covariate**	**Estimate (β)**	**SE**	***t* value**	**Pr(>|t|)**
Age	−0.000246	3.28 × 10^−5^	−7.5	6.80 × 10^−14^
Sex, F	−0.040726	1.15 × 10^−3^	−35.3	9.80 × 10^−273^
pc.0	0.006926	5.90 × 10^−4^	11.7	8.00 × 10^−32^
pc.1	−0.001214	5.80 × 10^−4^	−2.1	0.036
pc.2	−0.001546	5.80 × 10^−4^	−2.7	0.0077
pc.3	−0.000978	5.79 × 10^−4^	−1.7	0.091
pc.4	0.001443	5.82 × 10^−4^	2.5	0.013
Autosomal variance	0.640646	8.52 × 10^−4^	752.3	<×10^−300^
Hap H1 (203003)	0.000753	2.47 × 10^−3^	0.3	0.76
Hap. H2 (31389)	0.017434	3.86 × 10^−3^	4.5	6.10 × 10^−6^
Hap. H3 (28107)	0.082418	4.01 × 10^−3^	20.6	6.80 × 10^−94^
Hap. H4 (15052)	0.047945	5.11 × 10^−3^	9.4	6.30 × 10^−21^
Hap. H5 (37219)	0.018601	3.64 × 10^−3^	5.1	3.20 × 10^−7^
Hap. H6 (20240)	0.040591	4.53 × 10^−3^	9	3.40 × 10^−19^
Hap. HV (23855)	0.021313	4.26 × 10^−3^	5	5.80 × 10^−7^
Hap. J1 (76312)	0.092318	2.96 × 10^−3^	31.2	1.20 × 10^−213^
Hap. J2 (17812)	0.113978	4.77 × 10^−3^	23.9	3.70 × 10^−126^
Hap. K1 (71844)	0.134303	3.02 × 10^−3^	44.4	<×10^−300^
Hap. K2 (18034)	0.134744	4.75 × 10^−3^	28.3	1.10 × 10^−176^
Hap. T1 (21921)	0.148079	4.40 × 10^−3^	33.7	1.10 × 10^−248^
Hap. T2 (76650)	0.118618	2.96 × 10^−3^	40.1	<×10^−300^
Hap. U2 (12438)	0.134402	5.53 × 10^−3^	24.3	3.20 × 10^−130^
Hap. U4 (23358)	0.122405	4.29 × 10^−3^	28.5	1.10 × 10^−178^
Hap. U5 (84439)	0.120046	2.89 × 10^−3^	41.5	<×10^−300^
Hap. V (17675)	0.009295	4.78 × 10^−3^	1.9	0.052
Hap. X2 (14869)	0.049903	5.13 × 10^−3^	9.7	2.50 × 10^−22^
Hap. Other (115492)	0.079721	2.71 × 10^−3^	29.4	1.90 × 10^−190^

The impact of mitochondrial haplogroup was evaluated for the 19 most common haplogroups that were present in the population, each having at least 10,000 individuals, i.e., ~1% of the total population (Table 2). Mean MtHz differed between differing haplogroups. H1 was the most common haplogroup in the dataset (*n* = 203,003; 20.7%). Although the impact on MtHz was significant for many of the haplogroups, the magnitude of the MtHz difference was small, and individuals with T1 haplogroup (*n* = 21,921; 2.2%) had a mean MtHz that was only 0.0010 greater than H1 individuals (*P* = 1.1 × 10^−248^ in the null model). Within the population, mean MtHz significantly declined with increasing age. In addition, females had significantly lower mean MtHz than males. Age and sex were included as covariates in the GWAS model.

### Elimination of mitochondrial pseudogenes

**Fig. 3:**
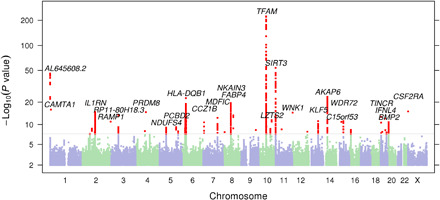
Manhattan plot of the initial association with mean MtHz.

We performed GWAS of mean MtHz. Initially, 37 loci reached genome-wide significance, *P* < 5 × 10^−8^, after adjusting for a genomic inflation factor of λ = 1.077 (Fig. 3 and fig. S2). However, an important potential confounder is the presence of nuclear mtDNA segments (NUMTs). NUMTs include partial, fragmented, complex, or complete copies of the mtDNA that have been retrotransposed into the nuclear genome (*17*, *18*). The direct hybridization of ostensibly mitochondrial probes to nuclear DNA from NUMTs will overestimate MtHz if the NUMT sequence contains alleles that vary from an individual’s true mtDNA sequence. Therefore, NUMTs have the potential to produce false-positive GWAS signals at SNPs, which are in linkage disequilibrium (LD).

An example of this is illustrated at rs1951197 (near *AKAP6*), which was associated with log mean MtHz (β = 0.0087, *P* = 7.81× 10^−26^). This position overlaps NUMT 474, which is polymorphic in Europeans (esv3633987) (fig. S3). NUMT 474 is 93% identical to positions 5583 to 6606 of the revised Cambridge Reference Sequence (rCRS) mtDNA (NC_012920.1). We tested the validity of the association of rs1951197 by repeating analysis after removing any position between mt.5583 and mt.6606 from the quantitation of MtHz (table S1). As expected, the association between rs1951197 and mean MtHz was no longer significant. Using a similar approach, we also eliminated the association of rs7728823, which overlaps NUMT 228, and rs571982832, which maps to a recently identified complex NUMT (*18*).

Because not all NUMTs are known or precisely located, we sought to identify other loci where apparent association was driven by cryptic NUMTs. We separately estimated mean MtHz after dividing the mitochondrial positions into three groups. We reasoned that nuclear positions would show marked differences in associations with these subsets of mitochondrial positions if NUMTs drove an artificial association. SNPs were removed from further analysis when the association was reduced below genome-wide significance when the analysis of heteroplasmy was based on a subregion. This identified four additional loci whose association was strongly region dependent (table S1), and these were also removed from the subsequent analyses.

### Genome-wide association identifies loci associated with MtHz

After the exclusion of NUMT-dependent positions, 30 loci initially had at least one position with genome-wide significance. To avoid evaluating poorly supported associations, 10 loci at which only a single imputed SNP exceeded genome-wide significance were removed from further analysis, leaving a final 20 loci associated with mean MtHz (Table 3 and fig. S4). Estimated from the array, the observed scale heritability of MtHz was 0.65% (SE = 0.1%, mean χ^2^ = 1.1914, intercept = 1.032) (*19*). The 20 loci accounted for 32% of the observed heritability (table S2). We confirmed the lack of dependence of these associations upon haplogroup by retesting the peak association with the inclusion of haplogroup in the model (table S3).

**Table 3:** Loci associated with mean MtHz. The positions (GRCh37/hg19 assembly) are ordered by *P* value, and those exceeding genome-wide significance are provided. The SNP with the smallest *P* value in each interval is given. Beta values are the per-allele effect of the B allele upon natural log mean MtHz value.

**Chromosome**	**Position**	**SNP**	**Alleles (A/B)**	**BAF**	**Beta**	**95% CI**	***P***	**Nearest** **gene(s)**	**Nearest gene** **function**
10q21.1	60,155,120	rs1049432	G/T	0.183	0.035	0.033–0.037	2 × 10^–223^	*TFAM*	mtDNA packaging factor
6p21.32	32,626,574	rs28539606	A/G	0.147	0.020	0.016–0.024	4 × 10^–23^	*HLA-DQB1*	Immune response
Xp22.3	1,413,667	rs28602228	C/T	0.371	0.008	0.006–0.010	8.4 × 10^–16^	*CSF2RA*	Signal transduction
2q13	113,876,498	rs4251979	C/T	0.733	−0.008	−0.010 to −0.006	1.2 × 10^–15^	*IL1RN*	Viral response
3p14.3	58,302,935	rs73081554	C/T	0.068	−0.014	−0.018 to −0.010	3.2 × 10^–14^	*RPP14*	Nuclear ribonuclease P component
19p13.3	5,555,098	rs12461806	A/G	0.913	−0.012	−0.015 to −0.009	1.8 × 10^–13^	*TINCR*	Noncoding RNA
19q13.2	39,737,576	rs370209610	C/T	0.981	−0.023	−0.029 to −0.016	6.3 × 10^–13^	*IFNL4*	Viral response
10q24.31	102,764,338	rs58678340	C/T	0.014	−0.026	−0.033 to −0.018	3.2 × 10^–12^	*TWNK*/ *MRPL43*	mtDNA helicase/ mitoribosome
15q21.3	54,107,732	rs200605061	D/I	0.121	0.009	0.007–0.012	6.9 × 10^–12^	*WDR72*	Regulator of membrane shape
13q22.1	73,690,621	rs7319964	A/T	0.539	−0.006	−0.008 to −0.004	7.4 × 10^–12^	*KLF5*	Nuclear DNA binding factor
20p12.3	7,014,445	rs2149642	C/T	0.775	0.007	0.005–0.009	1.5 × 10^–11^	*BMP2*	Bone morphogenetic protein
7p22.1	6,933,726	rs143803034	A/G	0.961	−0.015	−0.019 to −0.011	1.8 × 10^–11^	*CCZ1B*	Vacuolar fusion protein
5q11.2	52,832,775	rs10063311	C/G	0.224	−0.006	−0.008 to −0.004	9.4 × 10^–10^	*NDUFS4*	Structural component of complex I
2p11.2	87,831,354	rs145232625	C/T	0.257	−0.006	−0.008 to −0.004	2.0 × 10^–9^	*PLGLB2*	Plasminogen like protein
10q23.32	93,306,966	rs4933661	C/G	0.356	0.005	0.004–0.007	2.2 × 10^–9^	*HECTD2*	Ubiquitin ligase
16p13.13	11,143,355	rs758049676	D/I	0.432	0.005	0.003–0.007	4.1 × 10^–9^	*CLEC16A*	Promoter of antigen presentation
5q32	149,579,857	rs2286639	A/G	0.208	−0.006	−0.008 to −0.004	1.0 × 10^–8^	*SLC6A7*	Neurotransmitter transport
12q24.23	120,146,925	rs11064881	A/G	0.926	−0.009	−0.012 to −0.006	1.4 × 10^–8^	*CIT*/*PRKAB1*	Ser-Thr kinase/ AMP-kinase subunit
19q13.33	49,206,462	rs681343	C/T	0.484	0.005	0.003–0.006	2.0 × 10^–8^	*FUT2*/ *MAMSTR*	Fucosyltransferase/ transcription factor
2p13.2	72,256,404	rs11679052	C/G	0.578	−0.005	−0.006 to −0.003	5.0 × 10^–8^	*CYP26B1*	Retinoic acid metabolism

**Fig. 4:**
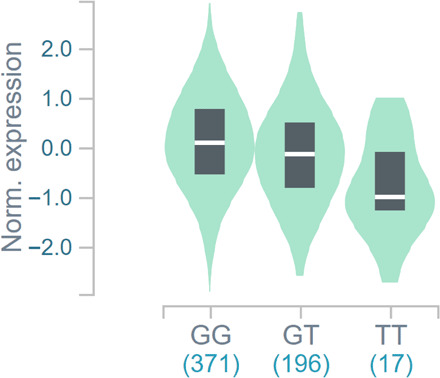
Association of rs1049432 with TFAM expression. Data for tibial artery expression are shown, and the numbers below the genotype are the sample size. *P* = 9.3 × 10^−30^. Normalized effect size = −0.33.

Of these candidates, three loci are proximal to four genes (*TFAM*, *TWNK*, *MRPL43*, and *NDUFS4*) with clear mitochondrial function and documented mitochondrial localization (*20*). The strongest association was identified at rs1049432 (*P* = 1.7 × 10^−223^), which is proximal to mitochondrial transcription factor A (*TFAM*). *TFAM* is an intriguing candidate with many interactions with mtDNA. TFAM was initially characterized as a mitochondrial transcription factor (*21*), but over time, numerous roles have emerged for TFAM in the maintenance and packaging of mtDNA (*22*). Previous tissue-specific expression quantitative trait loci analyses show that the T allele at rs1049432, which was associated with elevated MtHz, is significantly associated with lower *TFAM* expression (Fig. 4 and fig. S5). rs1049432 is noncoding and is correlated with rs1937 (p.Ser12Thr) (*r*^2^ = 0.44), which is the most commonly observed coding SNP in *TFAM* (MAF = 0.08).

Previous studies showed that rs11006126, which is proximal and in strong LD with our identified *TFAM* peak at rs1049432 (*r*^2^ = 0.96) and included in our credible set (rs11006126), is associated with mtDNA copy number in saliva and blood samples in two studies (*23*, *24*). We tested whether alterations in mtDNA copy number could potentially confound our findings on MtHz. We quantified the relative mtDNA copy number phenotype using intensity data from the genotyping array. We retested both the null model for MtHz and the association between mean MtHz and the candidate loci from the GWAS and found that the identified associations were independent of mtDNA copy number (tables S4 and S5).

Beyond *TFAM*, other genes proximal to associated SNPs have the potential to affect MtHz based on their known cellular activities. This includes rs58678340 near *C10orf42*, which encodes the mitochondrial helicase *Twinkle* (*TWNK*) and the mitochondrial ribosomal protein *MRPL43*. Notably, this SNP is in strong LD with the *TWNK* coding variant rs17113613 (*r*^2^ = 0.84; p.Val368Ile)*. TWNK* is part of the replication machinery of mtDNA, and inherited defects lead to syndromes with depleted or deleted mtDNA (*25*). *TWNK* protects against the emergence of mtDNA variation (*26*), suggesting a potential mechanism that associates changes in TWNK activity with MtHz.

MtHz was also associated with rs10063311, which is proximal to *NDUFS4*, a subunit of complex I of the electron transport chain. Although no clear link exists between the operation of the electron transport chain and mtDNA integrity, it was previously shown that the loss of complex V subunits affected mtDNA quantity (*27*).

In addition to these, *CLEC16A* and *PRKAB1* have potential functional links to the fidelity and stability of mtDNA replication despite the absence of mitochondrial localization by the encoded proteins. *CLEC16A*, identified from a GWAS of type 1 diabetes, regulates mitophagy through its interaction with NDRP1 and PARKIN (*28*). *PRKAB1* encodes a subunit of adenosine monophosphate–activated protein kinase, which has been implicated in a range of pathways that promote biogenesis and energy production within the mitochondria (*29*). This suggests that cytosolic and nuclear processes important for mitochondrial QC play a role in the regulation of MtHz.

Several genes with roles in immunity, including *HLA-DQB1*, *IL1RN*, *IFNL4*, and *FUT2*, were located proximal to SNPs associated with MtHz. Variations in immune system function may have a direct impact upon MtHz, but it is also possible that these variants may affect the ratio between cell types in the DNA sample. Further testing in other sample types would be required to confirm this association.

### Pathway and gene-based analysis

We evaluated our GWAS results using gene set analysis (MAGMA [v1.07]) and identified that the Gene Ontology biological pathway of urate transport was enriched for associations (*P* = 6.7 × 10^−7^) (*30*, *31*). The association remained significant after Bonferroni correction for 15,484 pathways. There were five genes within the set (*SLC22A13*, *SLC2A9*, *SLC17A1*, *SLC17A3*, and *SLC22A12*), but none were in loci that met GWAS significance. Gene-based results for *SLC17A1* and *SLC17A3* both met significance criteria (*P* = 4.1 × 10^−11^ and 8.4 × 10^−9^, respectively) and are adjacent in the genome, while the three other members of this gene set did not survive correction for multiple genes, suggesting that the signal may be driven by a single locus. In addition, the similarities between urate transport pathways and the mechanisms controlling MtHz are not intuitive.

### PheWAS analysis

Using phenome-wide association study (PheWAS) from 23andMe, we tested 19 SNPs for association with 1123 traits. Two hundred eighty-seven SNP-phenotype pairs met criteria for significance after applying a Bonferroni correction for the traits and SNPs evaluated (table S6). We focused on associations with SNPs most strongly associated with MtHz. For rs1049432 (*TFAM*), the T allele, associated with higher heteroplasmy, is associated with a reduced risk for polycystic ovarian syndrome (61,181 cases and 839,824 controls; odds ratio = 0.96, *P* = 6 × 10^−7^), an association that was not previously identified in a meta-GWAS for polycystic ovarian syndrome with 10,074 cases (*32*).

## DISCUSSION

We evaluated characteristics that affect the level of MtHz, using a large cohort with genotyping of Mt variants using arrays. There are several limitations to our work. First, the use of arrays has previously been validated for MtHz by comparison to allele-specific quantitative polymerase chain reaction but not with the exact array used in this study, and there may be noise present in the estimates of MtHz (*33*). An additional limitation of the study is that it does not evaluate all mitochondrial positions in its estimation of MtHz, but instead focuses on a subset of SNPs selected for their high call rate and appreciable BAF. Last, the tissue type used (saliva) may not be representative of all tissues for a trait affecting mtDNA.

We identified associations between age, sex, mitochondrial haplogroup, and the MtHz value. MtHz was lower with increasing age. This finding was unexpected, as previous studies have identified increasing MtHz with age. Sequencing of blood DNA from 356 individuals from the Framingham Heart Study found elevated MtHz at multiple positions across the genome with increasing age (*34*). Similarly, a study of 2077 Sardinians using leukocyte DNA also found increasing MtHz and copy number reduction with increasing age (*35*). One possible explanation for this discrepancy is that the type of tissue studied may affect the dynamics of MtHz and aging, and our observation may be specific to saliva. Another possibility is that our quantitation of MtHz across a larger number of positions may differ from previous studies, which used peaks of heteroplasmy or a smaller number of tested mtDNA sites in their analyses.

Variation in MtHz has not frequently been evaluated by sex. A study of urine samples from 235 patients with heteroplasmy for the pathogenic mt.3243A>G variant found that MtHz is higher in urine samples from males than females (*36*). However, a sequencing study using leukocyte DNA in 1035 individuals without mitochondrial disease did not identify significant sex differences (*5*). Similarly to age, this may reflect a property of the tissue evaluated. In addition, our study has higher power to detect smaller age- and sex-dependent differences in MtHz.

The possibility that females may generally have lower MtHz than males is intriguing from the perspective of mitochondrial inheritance, as the impact of male MtHz would be limited to the individual rather than risking the transmission of MtHz to subsequent generations. However, studies of somatic heteroplasmy cannot easily be extended to an understanding of the female germline.

We have identified 20 loci that are associated with levels of MtHz. Two of the loci are proximal to genes encoding proteins that are directly involved in the replication of mtDNA: the DNA binding *TFAM* and the mitochondrial helicase *TWNK*. Expression data suggest that variant rs1049432 at *TFAM* is associated with differences in *TFAM* expression in numerous tissues. One obvious question is whether haploinsufficiency of TFAM would be associated with increased mtDNA MtHz. It has been recently shown in a mouse model that reduced *TFAM* expression leads to a decline in the level of a pathogenic MtHz (*37*). Notably, this study showed that the consequences of a pathogenic variant were greater at lower copy number rather than high copy number regardless of the change in heteroplasmy, demonstrating that the copy number for the wild-type allele may be the controlling feature for disease phenotype. In the single report of patients with pathogenic variants in *TFAM*, homozygosity for a rare missense variant led to a loss of TFAM, mtDNA depletion, and a mitochondrial phenotype (*38*), but again, the impact of this TFAM loss on mitochondrial sequence fidelity is unknown.

TFAM is a multifunctional binding protein of mtDNA (*39*). As its name indicates, it was originally identified with its role in mitochondrial transcription, but TFAM also has diverse roles in mtDNA replication and in the overall compaction of the genome. An SNP proximal and in strong LD with our identified *TFAM* peak (*r*^2^ = 0.96) and included in our credible set (rs11006126) is associated with mtDNA copy number in both saliva and blood samples in two studies (*23*, *24*). The directionality of the observed effect on copy number is intriguing, as the allele associated with greater copy number in these studies is associated with increased MtHz. It is possible that the previously observed impacts of *TFAM* upon mtDNA copy number and our finding on heteroplasmy may be related. Cai *et al.* (*24*) observed that MtHz at the unstable dinucleotide repeat mt.514 to mt.523 is also associated with mtDNA copy number and may have been underpowered to observe this association at other loci.

In our analysis, we sought to exclude the impact of NUMTs. These blocks of mitochondrial sequence within the nuclear genome can inflate heteroplasmy estimates if their sequence is distinct from an individual’s mtDNA. This presents a potential problem in our study because NUMTs are in LD with proximal nuclear loci. We identified known NUMTs driving a false association with nuclear loci but also demonstrate that additional loci may be proximal to unidentified NUMTs. Our strategy for identifying these false associations may not be effective with very large “mega-NUMTs” that contain the full length of the mitochondrial genome; however, they are apparently uncommon in the population (*18*), limiting their impact.

The state of MtHz is an important property of organellar genomes that affects the emergence of novel combinations of polymorphisms and plays a crucial role in the penetrance and severity of disease due to pathogenic variants. Heteroplasmic variants can emerge both somatically and are altered by germline transmission. In this study, we evaluated a large sample to identify nuclear-encoded variants and nearby genes that influence heteroplasmy. Our results show that nuclear variants proximal to genes required for mtDNA replication (*TFAM* and *TWNK*) and others associated with mitochondrial capacity and QC (*CLEC16A* and *PRKAB1*) are associated with MtHz, potentially based on their role in maintaining replicative fidelity within the mitochondrion.

## MATERIALS AND METHODS

### Quality control

#### Selection of mtDNA variants

We performed QC of mtDNA variants in a subset of randomly selected 278,196 individuals. Variants were pruned to remove those that were noninformative. There were initially 3,287 V4 mtDNA variants, which we pruned to 641 variants by retaining variants meeting the criteria of MAF > 0.001, biallelic and platform genotype call rate > 99%. We partitioned the per-variant data into two BAF groups for the remainder of the QC process (“low BAF” and “high BAF” groups, with the former defined as BAF ≤ 0.5 and the latter defined as having BAF > 0.5) to account for the arbitrary assignment of the value of unity to one of the two alleles at each biallelic SNP. Because both alleles are present within the population, the maximal observable value for heteroplasmy is 0.5.

We applied two additional filtering steps to each variant separately in each BAF group. The first filter was related to the log_2_ R ratio (LRR) and was designed to remove samples with low intensity reflecting issues with probe binding. We computed the pooled SD of the LRR across the high and low BAF groups, and sample values that were below a threshold of three pooled SDs below each BAF group’s mean LRR were removed. We used the pooled SD to ensure similar threshold filtering in a majority of cases, as very small BAF groups with large variance will have less effective removal of samples with low LRR. We retained mtDNA variants with <1% of samples removed with this LRR threshold filter in both BAF groups.

The next filter was designed to remove poorly performing assays, which had limited discrimination of the A and B alleles that disrupted the expected clustering of homoplasmic data points for most individuals within the dataset. mtDNA variants where ≥10% of samples had ≥20% heteroplasmy (with minimum 10 samples in each group) were removed from the analysis.

There were 356 mtDNA variants remaining after the above. An additional 29 variants were then excluded from the hypervariable regions (positions in the following ranges: 57 to 372, 438 to 574, and 16024 to 16383), producing a final set of 326 mtDNA variants. Hypervariable regions were removed from the analysis because of concerns about hybridization to the array due to variability at sequences proximal to the tested position.

#### Sample QC

Samples were removed entirely from analysis if they were missing more than 5% of the data following variant QC.

#### Selection of autosomal SNPs used in autosomal variance detection

We selected a high-quality set of autosomal SNPs available on all 23andMe genotyping chips in Europeans with criteria MAF ≥ 0.1 and genotype call rate > 99.98%, leaving 8205 variants for further analysis. We then randomly sampled 10 SNPs per autosome to produce a final set of 220 autosomal SNPs. We then computed the autosomal equivalent of the mtDNA heteroplasmy measure, or “autosomal variance,” by computing the deviation of each sample’s BAF from their expected value of 0 or 1, across the subset of these variants that they had homozygous calls for. We used log-transformed autosomal variance (+ 1 × 10^−6^, as the minimum autosomal variance value was 0) as a covariate in the GWAS.

#### mtDNA copy number analysis

The mean LRR of the 326 evaluated mitochondrial positions was used to quantify the relative copy number between samples. We used the LRR cutoffs described above to remove low-intensity outliers caused by poor hybridization.

### Phenome-wide association study

Nineteen sentinel variants (of 20 initial candidate variants) were available for use in a PheWAS analysis with 1123 phenotypes in the 23andMe database, which comprised the comprehensive set for an internal large-scale GWAS run in 2018. The *P* values reported in the PheWAS study are unadjusted.

### Genome-wide association study

The GWAS model used phenotype natural log-transformed mean heteroplasmy across 326 variants in 982,072 unrelated European ancestry samples genotyped on the V4 array, with covariates age, sex, principal components 1 to 5, and natural log-transformed autosomal variance. We tested association with 57,525,634 imputed variants. All individuals included in the analyses provided informed consent and answered surveys online according to our human subject protocol, which was reviewed and approved by Ethical&Independent Review Services, a private institutional review board (http://www.eandireview.com).

DNA extraction and genotyping were performed on saliva samples by National Genetics Institute, a Clinical Laboratory Improvement Amendments–licensed clinical laboratory and a subsidiary of Laboratory Corporation of America. The platform was a fully customized array with additional coverage of lower-frequency coding variation and about 570,000 SNPs. Samples that failed to reach 98.5% call rate were reanalyzed. Individuals whose analyses failed repeatedly were recontacted by 23andMe customer service to provide additional samples.

For our standard GWAS, we restrict participants to a set of individuals who have a specified ancestry determined through an analysis of local ancestry (*40*). Briefly, our algorithm first partitions phased genomic data into short windows of about 300 SNPs. Within each window, we use a support vector machine (SVM) to classify individual haplotypes into one of 31 reference populations (www.23andme.com/ancestry-composition-guide/). The SVM classifications are then fed into a hidden Markov model (HMM) that accounts for switch errors and incorrect assignments and gives probabilities for each reference population in each window. Last, we used simulated admixed individuals to recalibrate the HMM probabilities so that the reported assignments are consistent with the simulated admixture proportions. The reference population data are derived from public datasets (the Human Genome Diversity Project, HapMap, and 1000 Genomes), as well as 23andMe customers who have reported having four grandparents from the same country. European ancestry was defined as European > 0.9.

A maximal set of unrelated individuals was chosen for each analysis using a segmental identity-by-descent (IBD) estimation algorithm (*41*). Individuals were defined as related if they shared more than 700-centimorgan IBD, including regions where the two individuals share either one or both genomic segments IBD. This level of relatedness (roughly 20% of the genome) corresponds approximately to the minimal expected sharing between first cousins in an outbred population. When selecting individuals for case-control phenotype analyses, the selection process is designed to maximize case sample size by preferentially retaining cases over controls. Specifically, if both an individual case and an individual control are found to be related, then the case is retained in the analysis.

Imputation panels created by combining multiple smaller panels have been shown to give better imputation performance than the individual constituent panels alone (*42*). To that end, we combined the May 2015 release of the 1000 Genomes Phase 3 haplotypes (*43*) with the UK10K imputation reference panel (*44*) to create a single unified imputation reference panel. To do this, multiallelic sites with N alternate alleles were split into N separate biallelic sites. We then removed any site whose minor allele appeared in only one sample. For each chromosome, we used Minimac3 (*45*) to impute the reference panels against each other, reporting the best-guess genotype at each site. This gave us calls for all samples over a single unified set of variants. We then joined these together to get, for each chromosome, a single file with phased calls at every site for 6285 samples. Throughout, we treated structural variants and small indels in the same way as SNPs.

In preparation for imputation, we split each chromosome of the reference panel into chunks of no more than 300,000 variants, with overlaps of 10,000 variants on each side. We used a single batch of 10,000 individuals to estimate Minimac3 imputation model parameters for each chunk.

To generate phased participant data for the v1 to v4 platforms, we used an internally developed tool, Finch, which implements the Beagle graph–based haplotype phasing algorithm (*46*), modified to separate the haplotype graph construction and phasing steps. Finch extends the Beagle model to accommodate genotyping error and recombination to handle cases where there are no consistent paths through the haplotype graph for the individual being phased. We constructed haplotype graphs for all participants from a representative sample of genotyped individuals and then performed out-of-sample phasing of all genotyped individuals against the appropriate graph. For the X chromosome, we built separate haplotype graphs for the non-pseudoautosomal region and each pseudoautosomal region, and these regions were phased separately. For the 23andMe participants genotyped on the v5 array, we used a similar approach, but using a new phasing algorithm, Eagle2 (*47*). We imputed phased participant data against the merged reference panel using Minimac3, treating males as homozygous pseudo-diploids for the non-pseudoautosomal region.

We compute association test results for the genotyped and the imputed SNPs. For case-control phenotypes, we compute association by logistic regression assuming additive allelic effects. For tests using imputed data, we use the imputed dosages rather than best-guess genotypes. As standard, we include covariates for age, gender, the top five principal components to account for residual population structure, and indicators for genotype platforms to account for genotype batch effects. The association test *P* value we report is computed using a likelihood ratio test, which, in our experience, is better behaved than a Wald test on the regression coefficient. For quantitative traits, association tests are performed by linear regression. Results for the X chromosome are computed similarly, with male genotypes coded as if they were homozygous diploid for the observed allele.

A principal components analysis was performed independently for each ancestry, using ~65,000 high-quality genotyped variants. It was computed on a subset of participants randomly sampled across all the genotyping platforms (1 million participants were used for European). Principal component scores for participants not included in the analysis were obtained by projection, combining the eigenvectors of the analysis and the SNP weights.

### Genotype-Tissue Expression

The data used for the analyses described in this manuscript were obtained from the Genotype-Tissue Expression (GTEx) Portal on 30 Oct 2020 v8. The GTEx Project was supported by the Common Fund of the Office of the Director of the National Institutes of Health and by National Cancer Institute, National Human Genome Research Institute, National Heart, Lung, and Blood Institute, National Institute on Drug Abuse, National Institute of Mental Health, and National Institute of Neurological Disorders and Stroke.

## Supplementary Material

http://advances.sciencemag.org/cgi/content/full/7/12/eabe7520/DC1

Adobe PDF - abe7520_SM.pdf

Nuclear genome-wide asociations with mitochondrial heteroplasmy

## SUPPLEMENTARY MATERIALS

Supplementary material for this article is available at http://advances.sciencemag.org/cgi/content/full/7/12/eabe7520/DC1

## Appendix

View/request a protocol for this paper from *Bio-protocol*.
