# Supplementary material for: Nuclear genome-wide associations with mitochondrial heteroplasmy
Source: Sci Adv. 2021 Mar 17;7(12):eabe7520. doi: 10.1126/sciadv.abe7520 (PMC7968846; doi:10.1126/sciadv.abe7520)
Supplement: http://advances.sciencemag.org/cgi/content/full/7/12/eabe7520/DC1 [file supp_7_12_eabe7520__7.12.eabe7520.DC1.html]

Science Advances | Science AdvancesAAASSearchScience AdvancesMenu

## Supplementary Materials

# Nuclear genome-wide associations with mitochondrial heteroplasmy

Priyanka Nandakumar, Chao Tian, Jared O’Connell, 23andMe Research Team, David Hinds, Andrew D. Paterson, Neal Sondheimer

Download Supplement

**This PDF file includes:**

- Members of the 23andMe Research Team
- Figs. S1 to S5
- Tables S1 to S6

**Files in this Data Supplement:**

- Adobe PDF - abe7520\_SM.pdf
