# Supplementary material for: Nuclear genome-wide associations with mitochondrial heteroplasmy
Source: Sci Adv. 2021 Mar 17;7(12):eabe7520. doi: 10.1126/sciadv.abe7520 (PMC7968846; doi:10.1126/sciadv.abe7520)
Supplement: http://advances.sciencemag.org/cgi/content/full/7/12/eabe7520/DC1 [file supp_7_12_eabe7520__abe7520_SM.pdf]

Science Advances
AAAS

advances.sciencemag.org/cgi/content/full/7/12/eabe7520/DC1

Supplementary Materials for

Nuclear genome-wide associations with mitochondrial heteroplasmy

Priyanka Nandakumar, Chao Tian, Jared O'Connell, 23andMe Research Team, David Hinds*, Andrew D. Paterson, Neal Sondheimer*

*Corresponding author. Email: neal.sondheimer@sickkids.ca (N.S.); dhinds@23andme.com (D.H.)

Published 17 March 2021, *Sci. Adv.* **7**, eabe7520 (2021)
DOI: 10.1126/sciadv.abe7520

This PDF file includes:

Members of the 23andMe Research Team
Figs. S1 to S5
Tables S1 to S6

Members of the 23andMe Research Team are: Michelle Agee, Stella Aslibekyan, Adam Auton, Elizabeth Babalola, Robert K. Bell, Jessica Bielenberg, Katarzyna Bryc, Emily Bullis, Briana Cameron, Daniella Coker, Gabriel Cuellar Partida, Devika Dhamija, Sayantan Das, Sarah L. Elson, Teresa Filshtein, Kipper Fletez-Brant, Pierre Fontanillas, Will Freyman, Pooja M. Gandhi, Karl Heilbron, Barry Hicks, Karen E. Huber, Ethan M. Jewett, Yunxuan Jiang, Aaron Kleinman, Katelyn Kukar, Keng-Han Lin, Maya Lowe, Marie K. Luff, Jennifer C. McCreight, Matthew H. McIntyre, Kimberly F. McManus, Steven J. Micheletti, Meghan E. Moreno, Joanna L. Mountain, Sahar V. Mozaffari, Elizabeth S. Noblin, Aaron A. Petrakovitz, G. David Poznik, Anjali J. Shastri, Janie F. Shelton, Jingchunzi Shi, Suyash Shringarpure, Vinh Tran, Joyce Y. Tung, Xin Wang, Wei Wang, Catherine H. Weldon, and Peter Wilton.

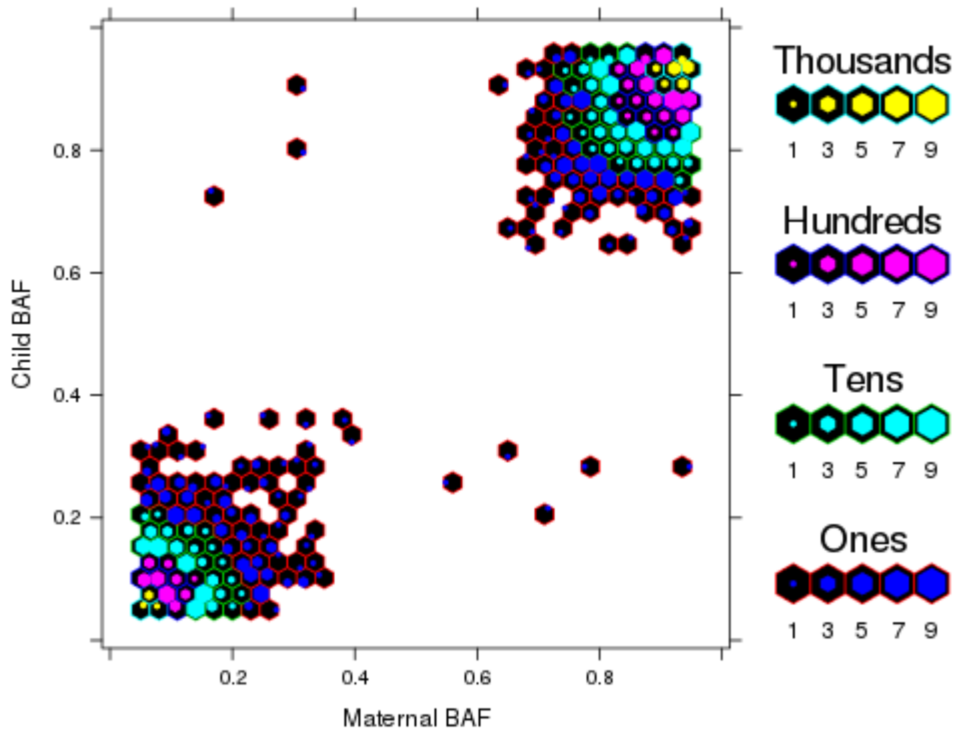

Fig. S1.

All heteroplasmic positions, where both the mother had >5% MtHz, are shown, plotted by both maternal and child BAF showing the predicted concordance of MtHz between mother and child.

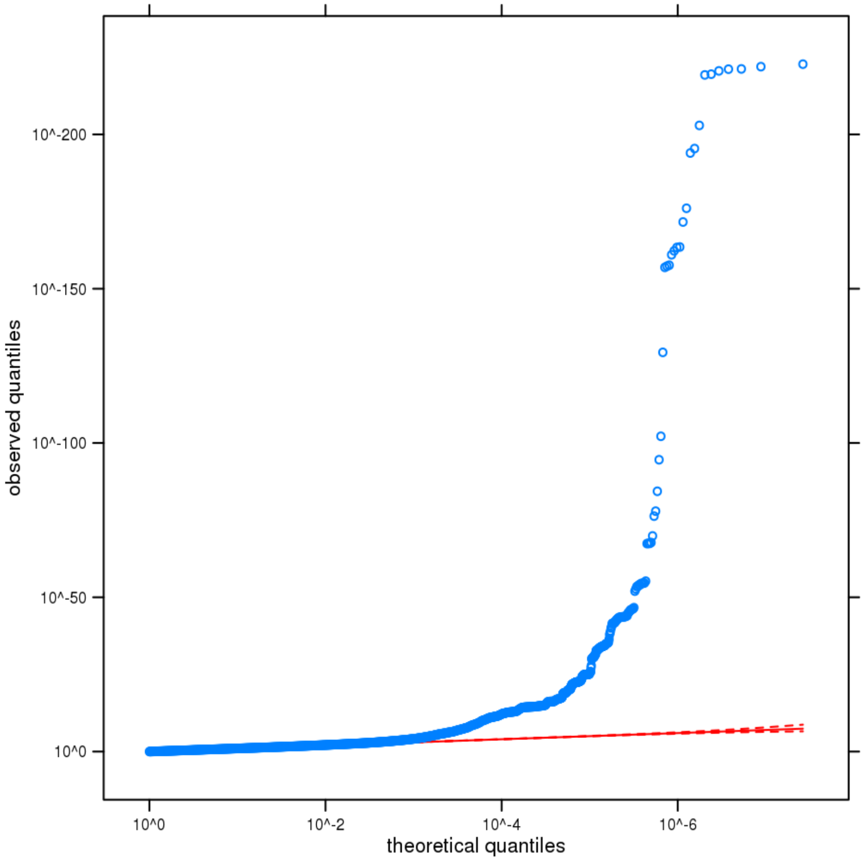

Fig. S2.

QQ plot. Red line indicates the null of no association. SNP association results presented in Table 3 are adjusted for λ=1.077

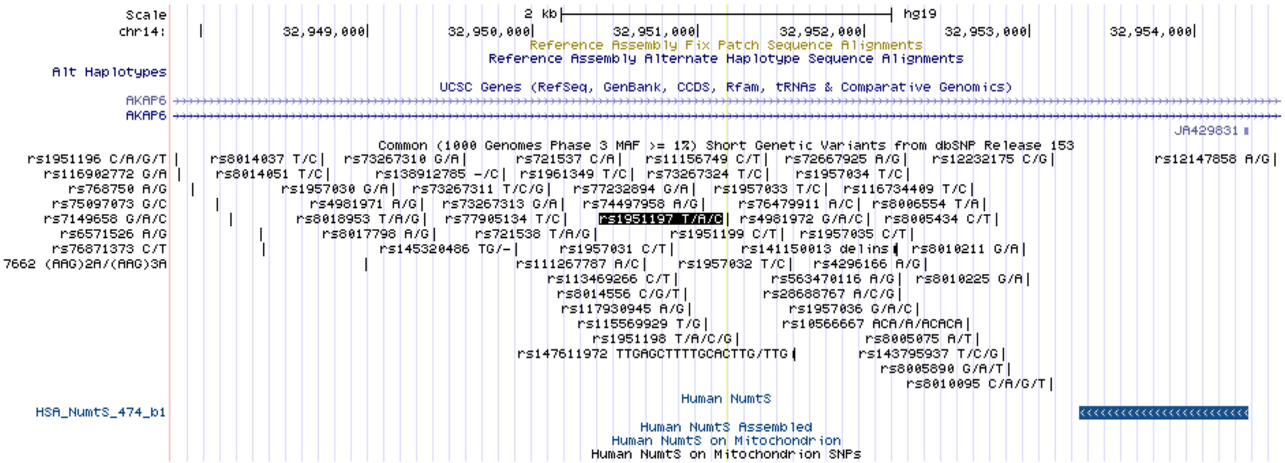

Fig. S3.

NUMT 474 overlap of rs1951197. The peak of association within AKAP6 lies within an intron that contains NUMT 474. View is from UCSC Genome Browser hg19.

Figure S4 – The following pages show the regional association plots for the 20 peak loci.

3.1 rs1049432: [TFAM]

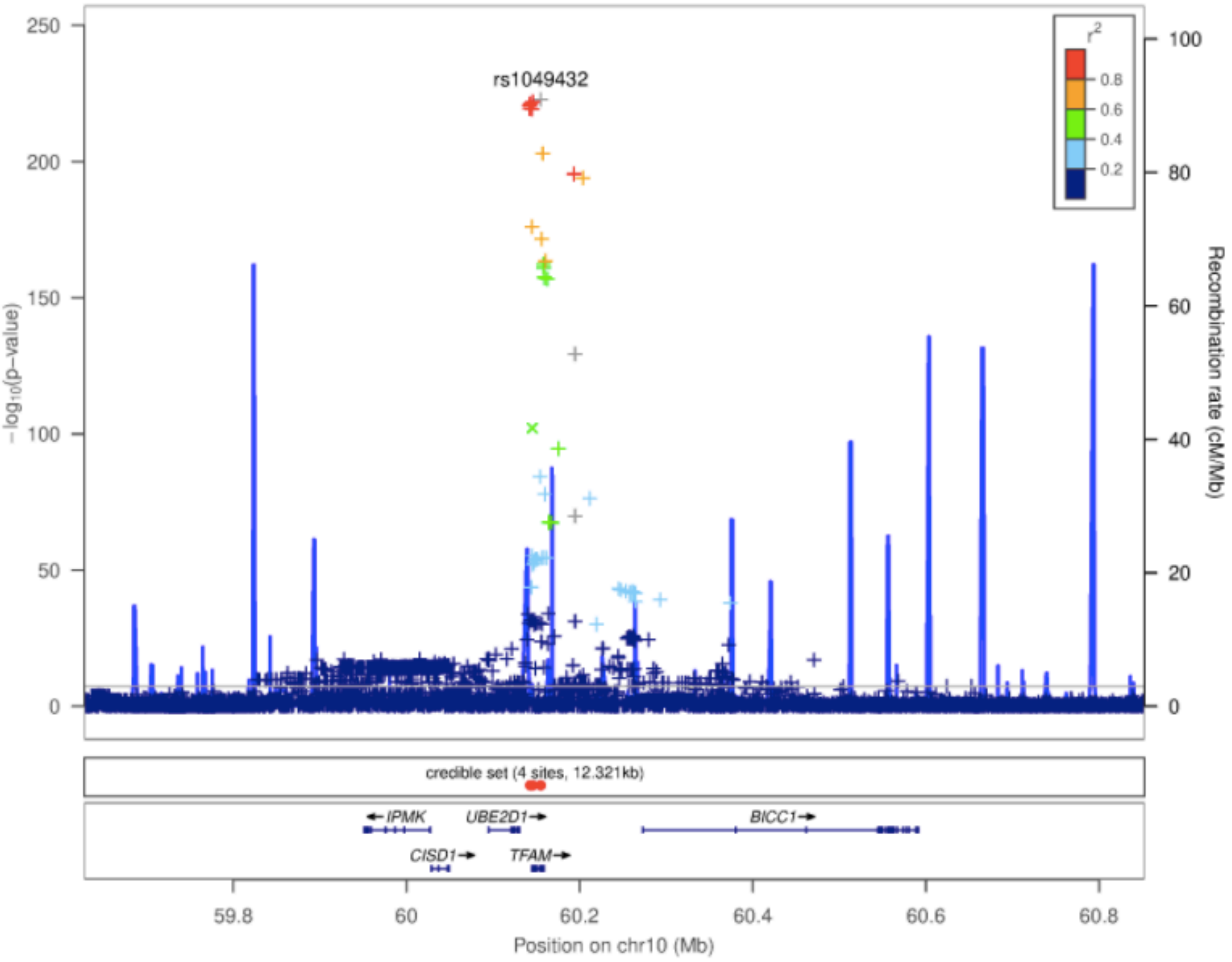

3.5 rs28539606: HLA-DQA1–[]HLA-DQB1

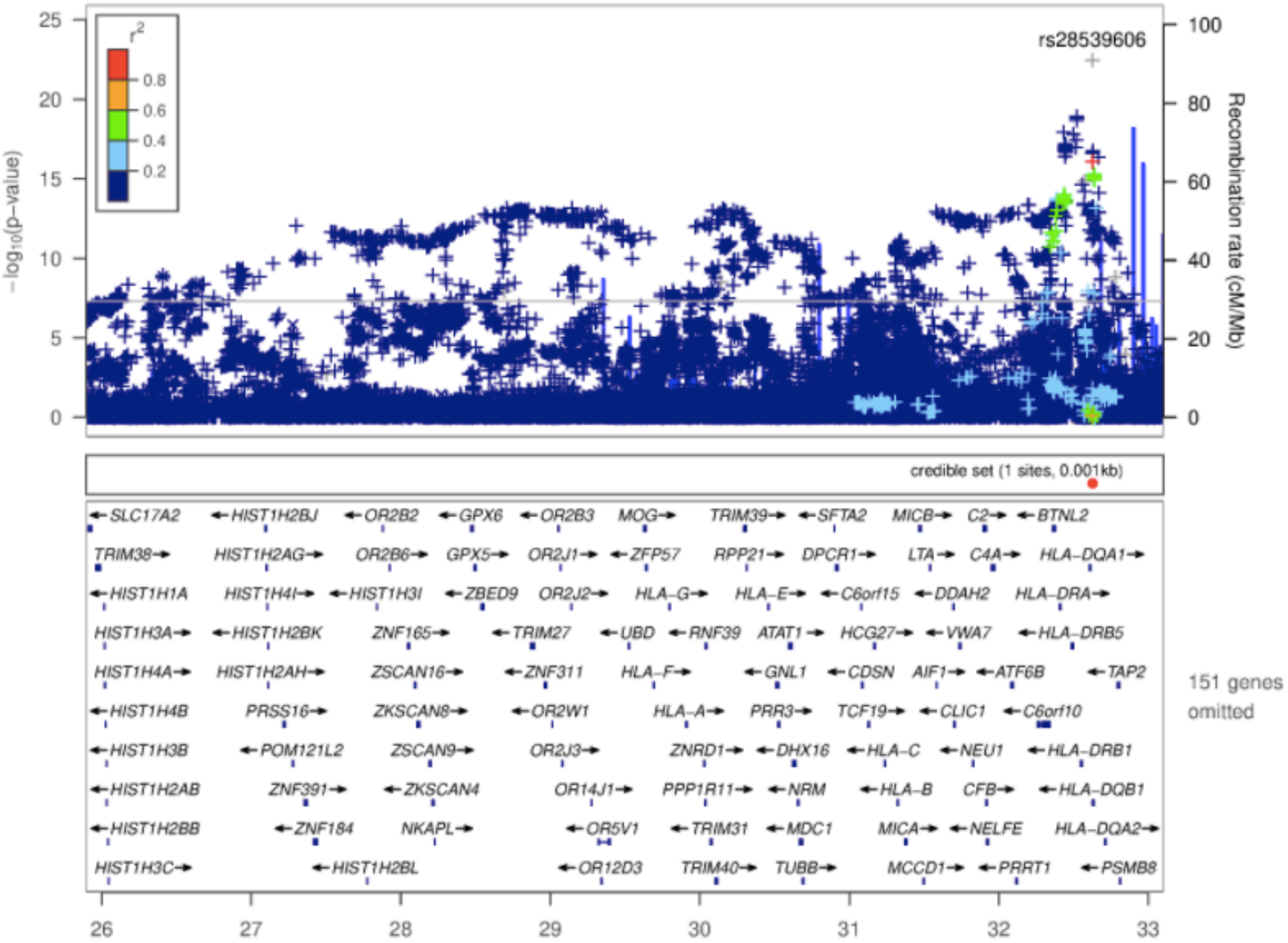

3.9 rs4251979: [IL1RN]

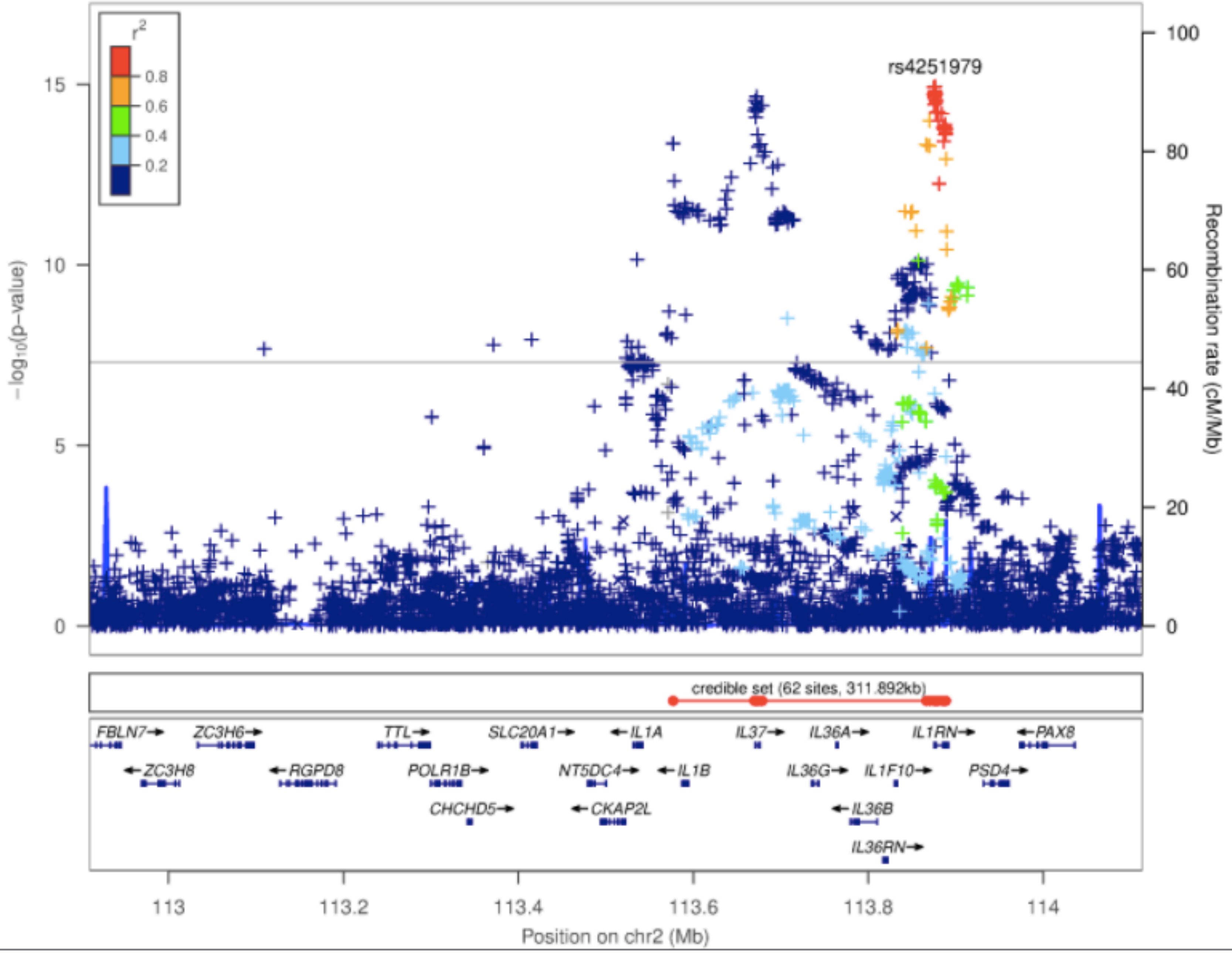

3.12 rs73081554: [RPP14,RP11-80H18.3]

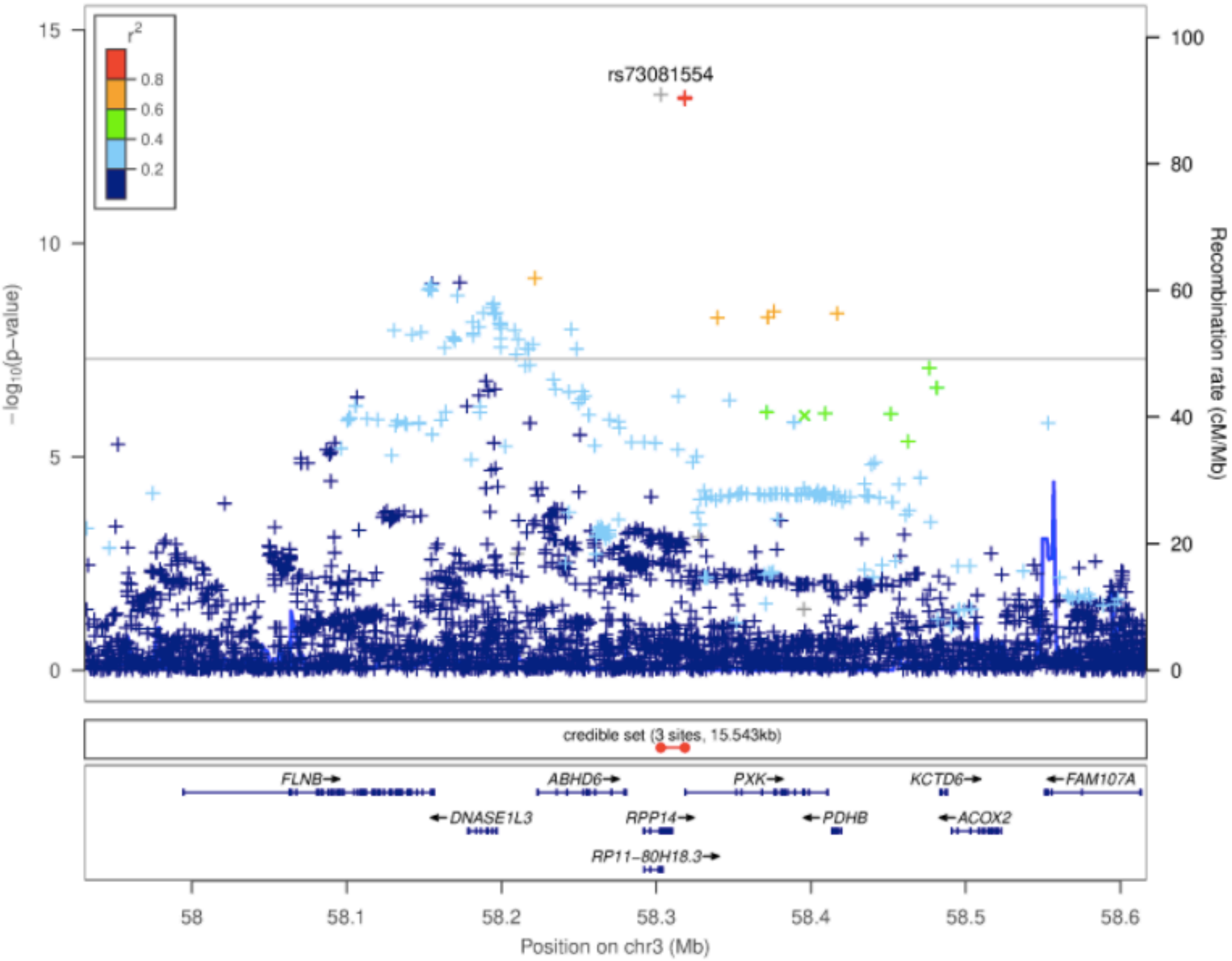

3.14 rs12461806: ZNRF4–[]-TINCR

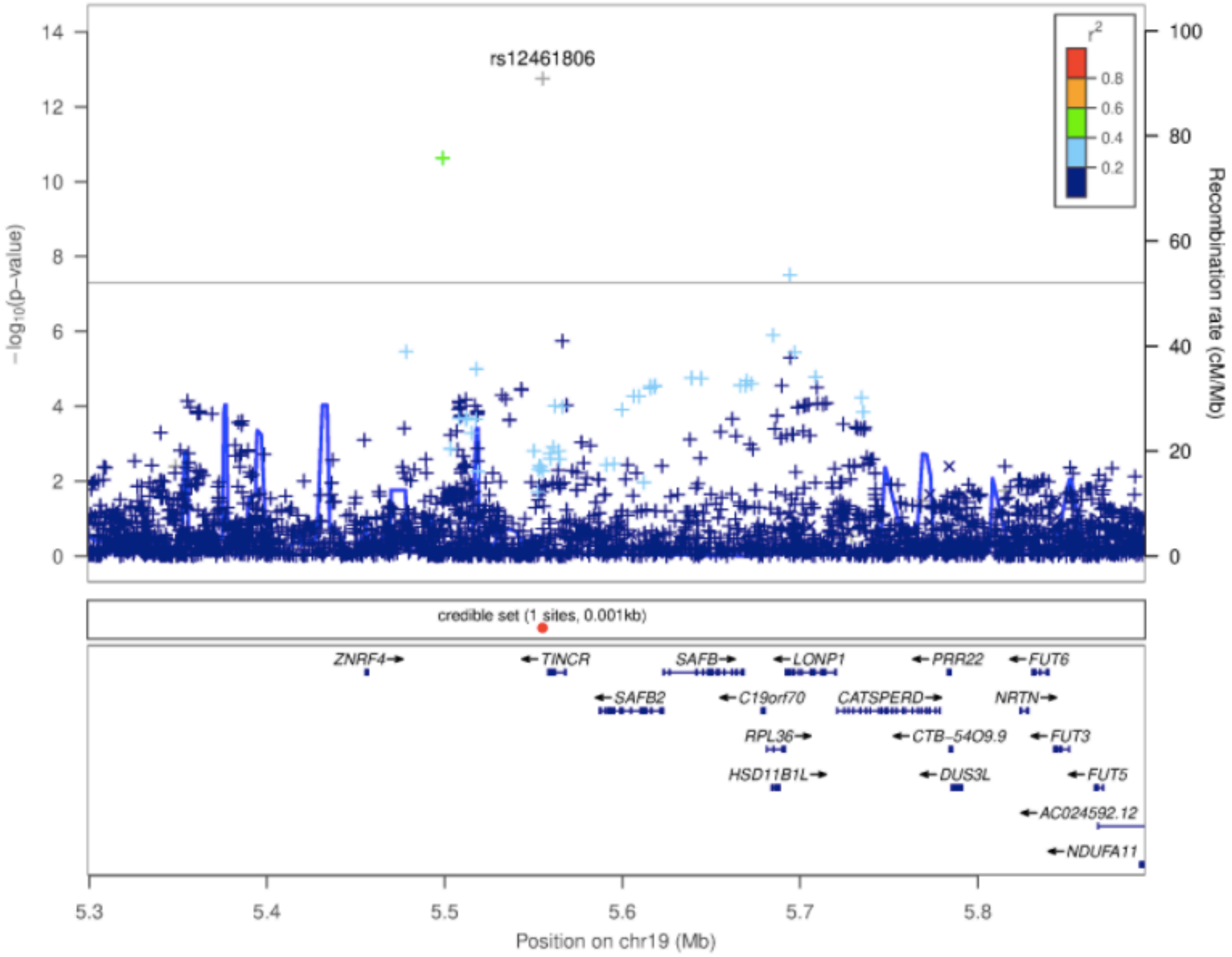

3.16 rs370209610: [IFNL4]

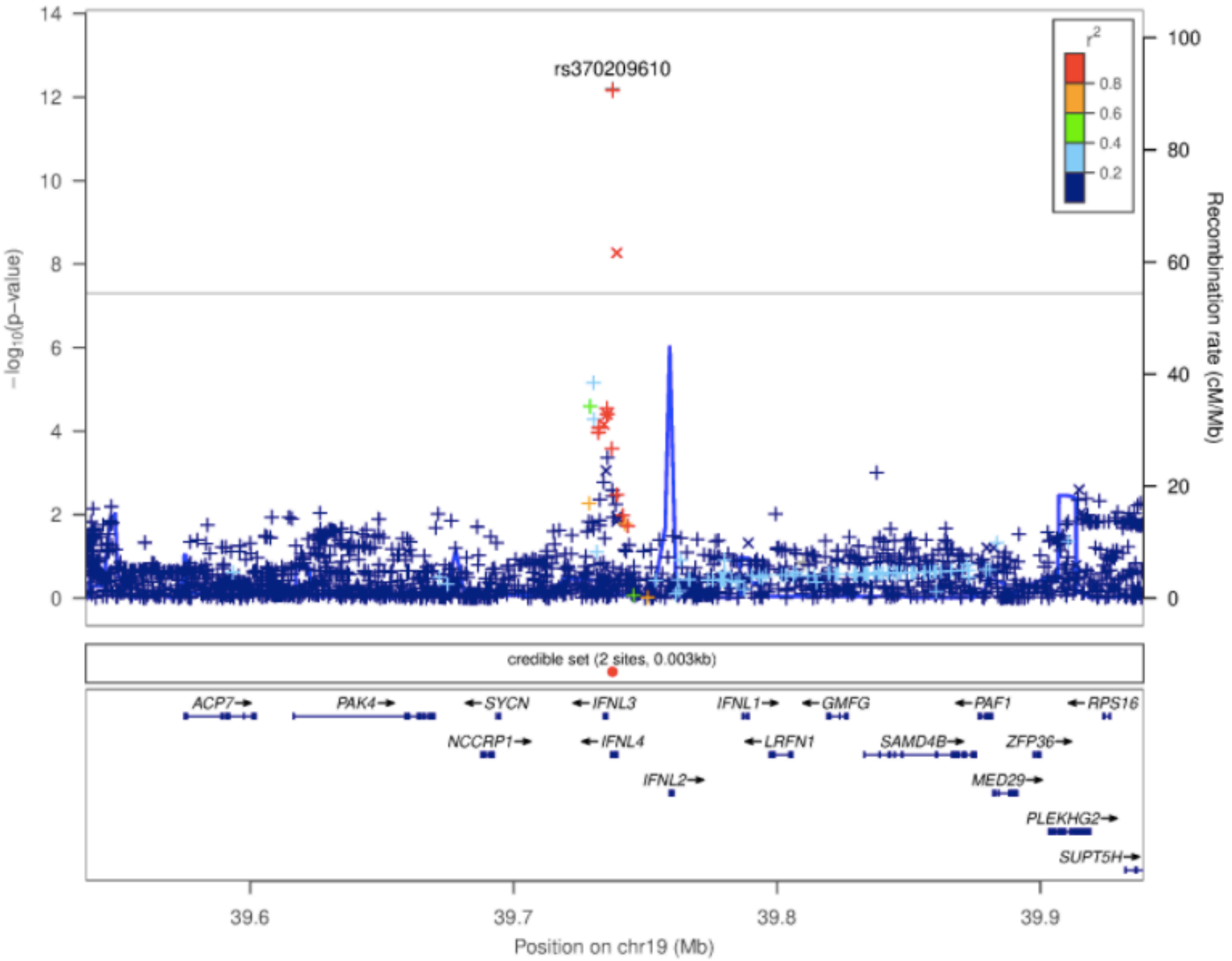

3.17 rs58678340: [LZTS2]

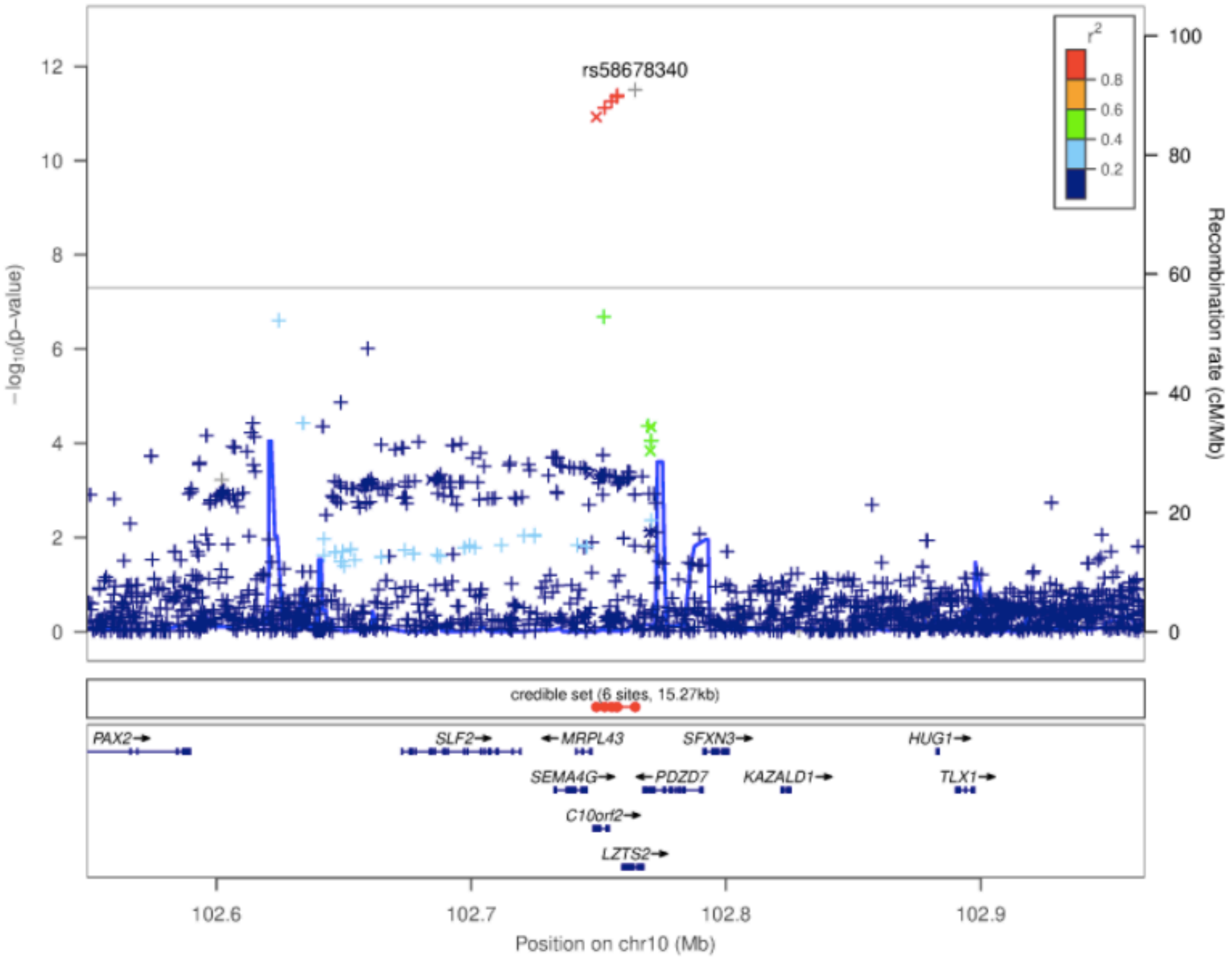

3.18 rs200605061:GTCAA: WDR72–[]—UNC13C

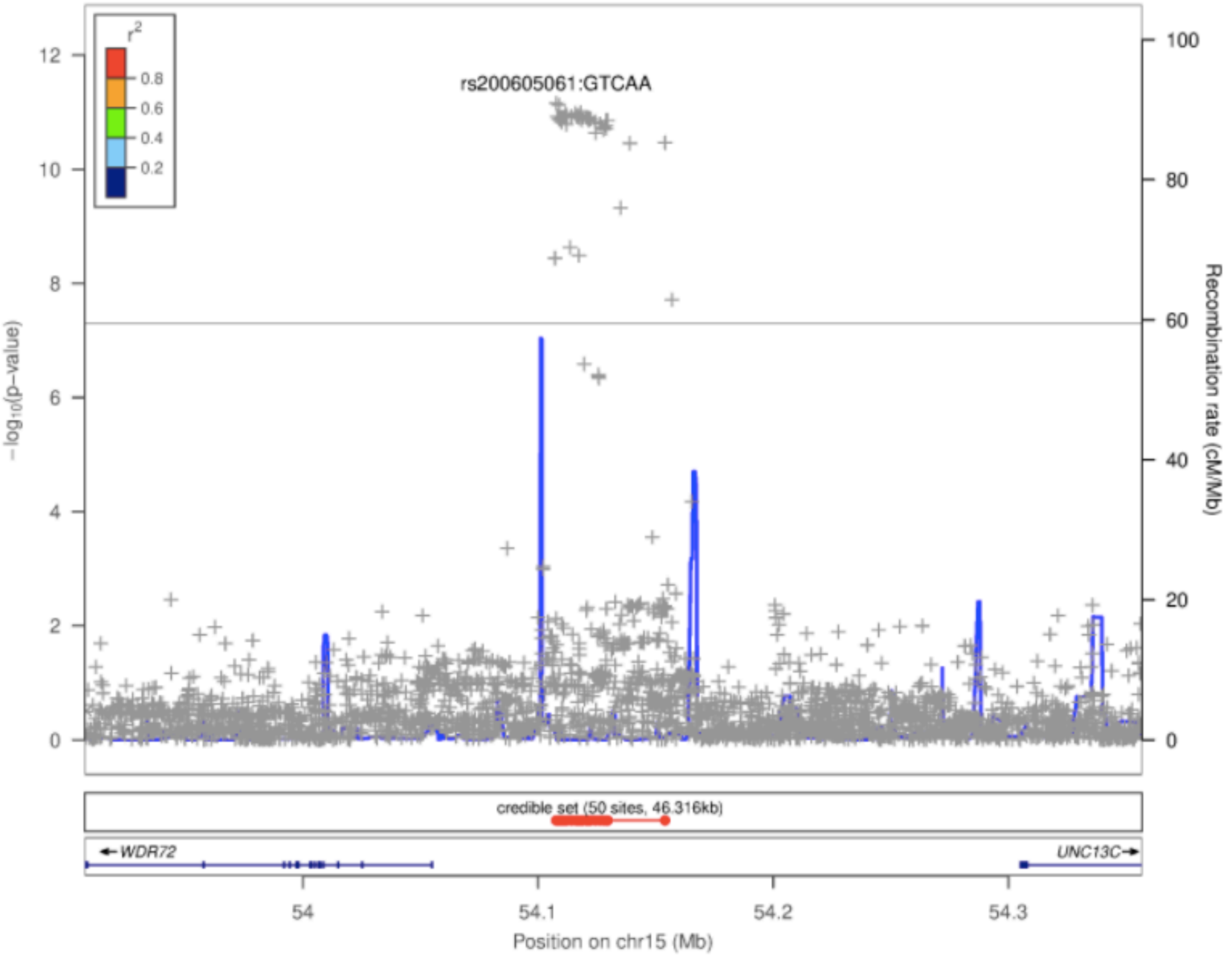

3.19 rs7319964: KLF5–[]—KLF12

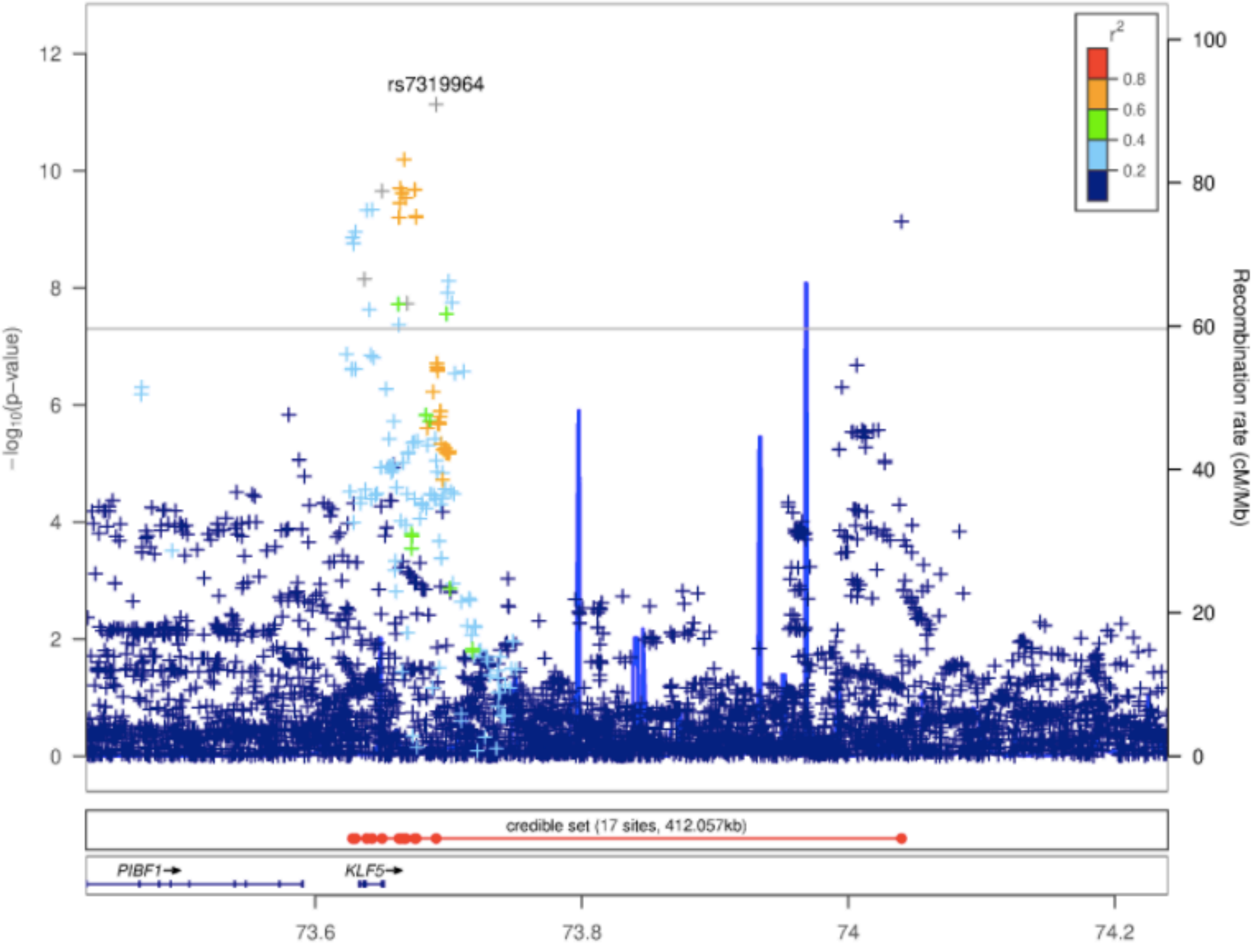

3.22 rs2149642: BMP2—[]—HAO1

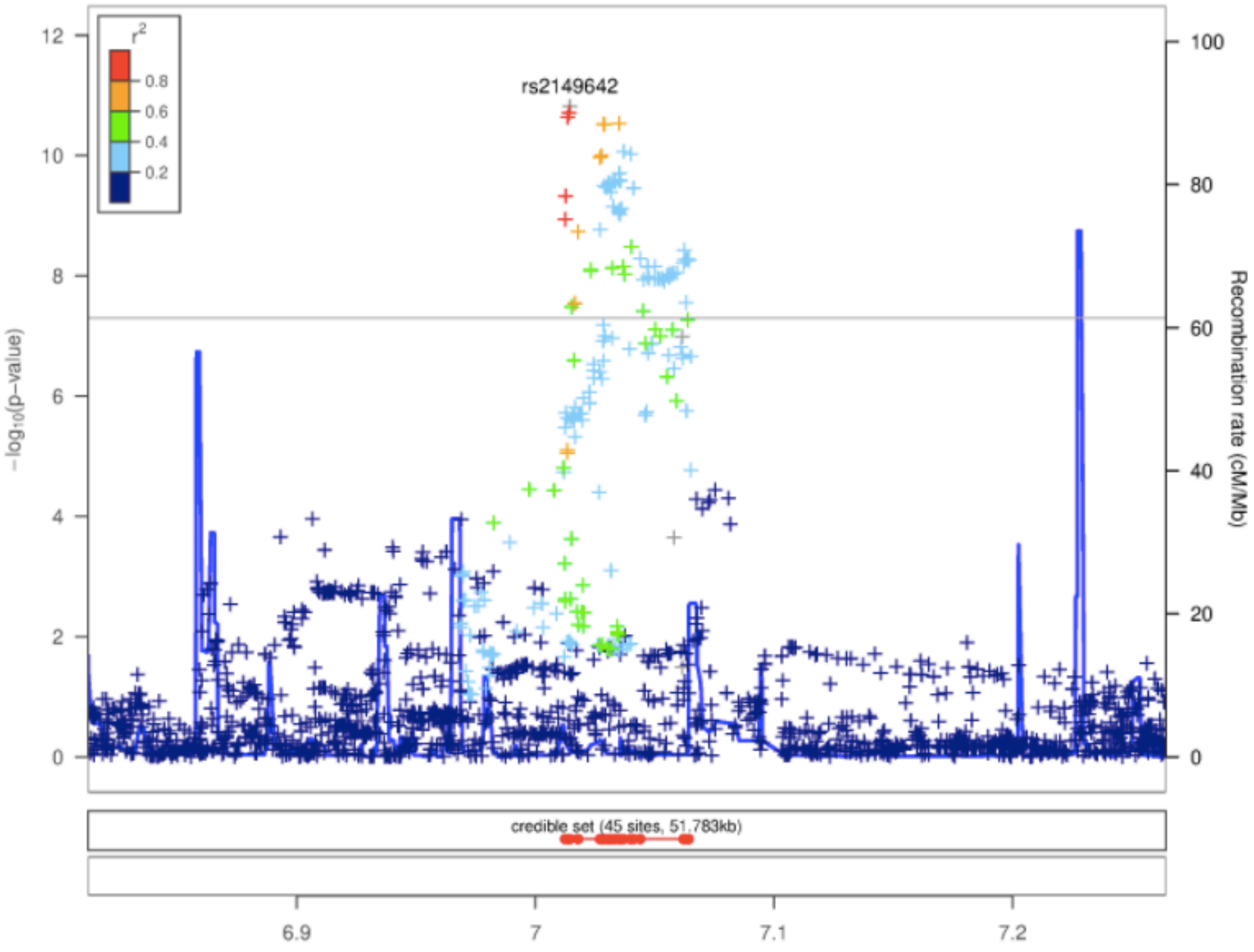

3.23 rs143803034: CCZ1B–[]–C1GALT1

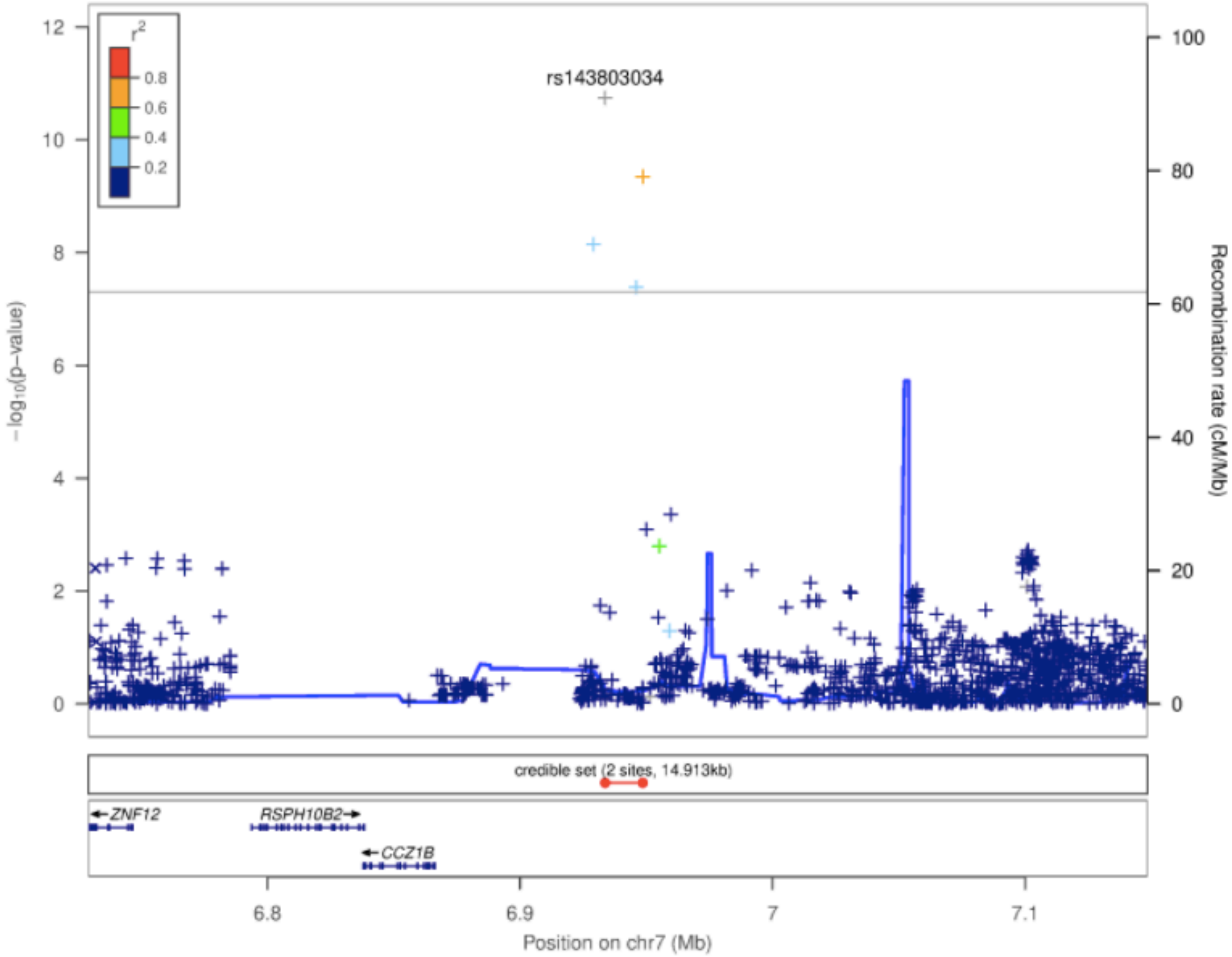

3.25 rs10063311: FST–[]–NDUFS4

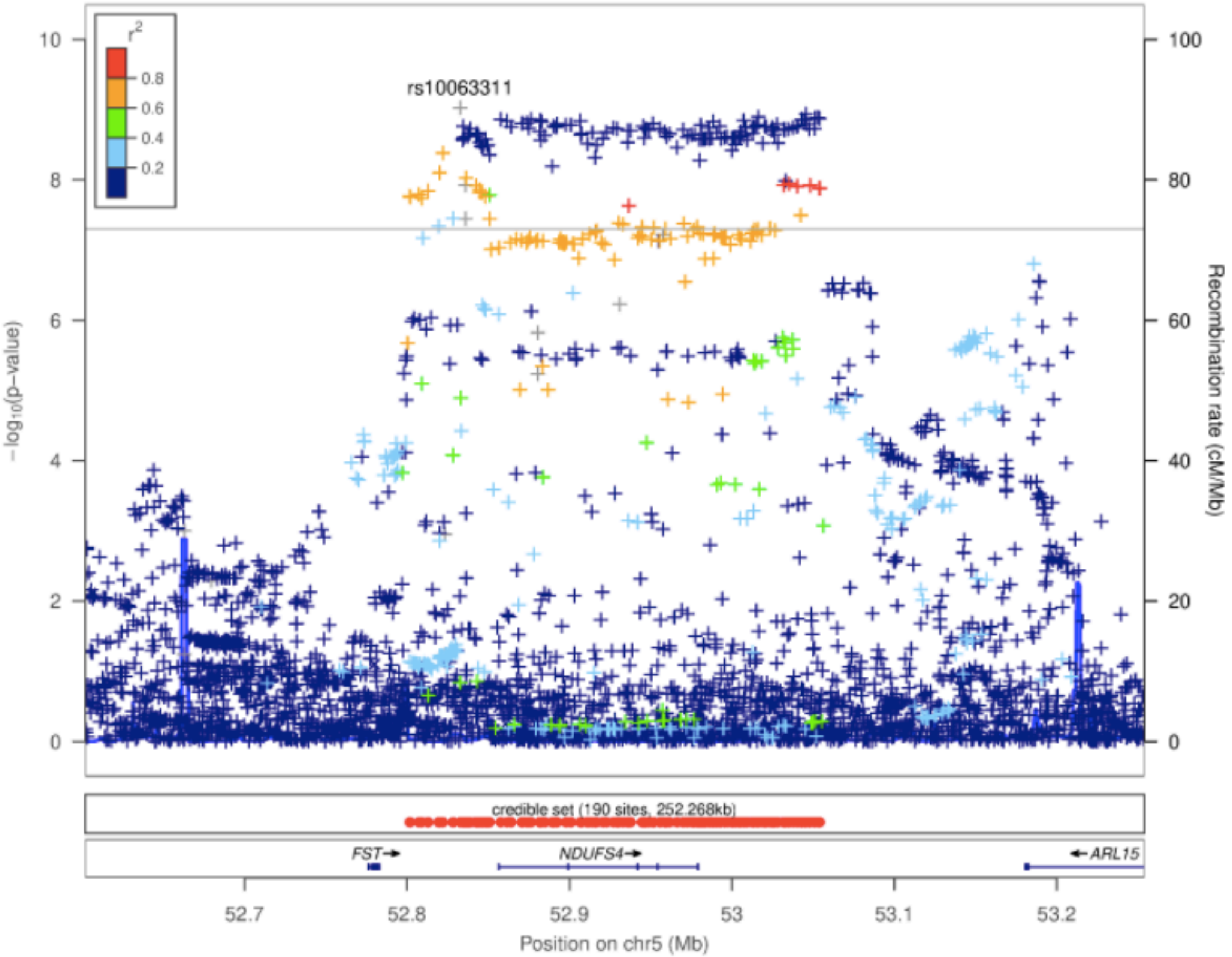

3.26 rs145232625: IGKV3OR2-268—[]—PLGLB2

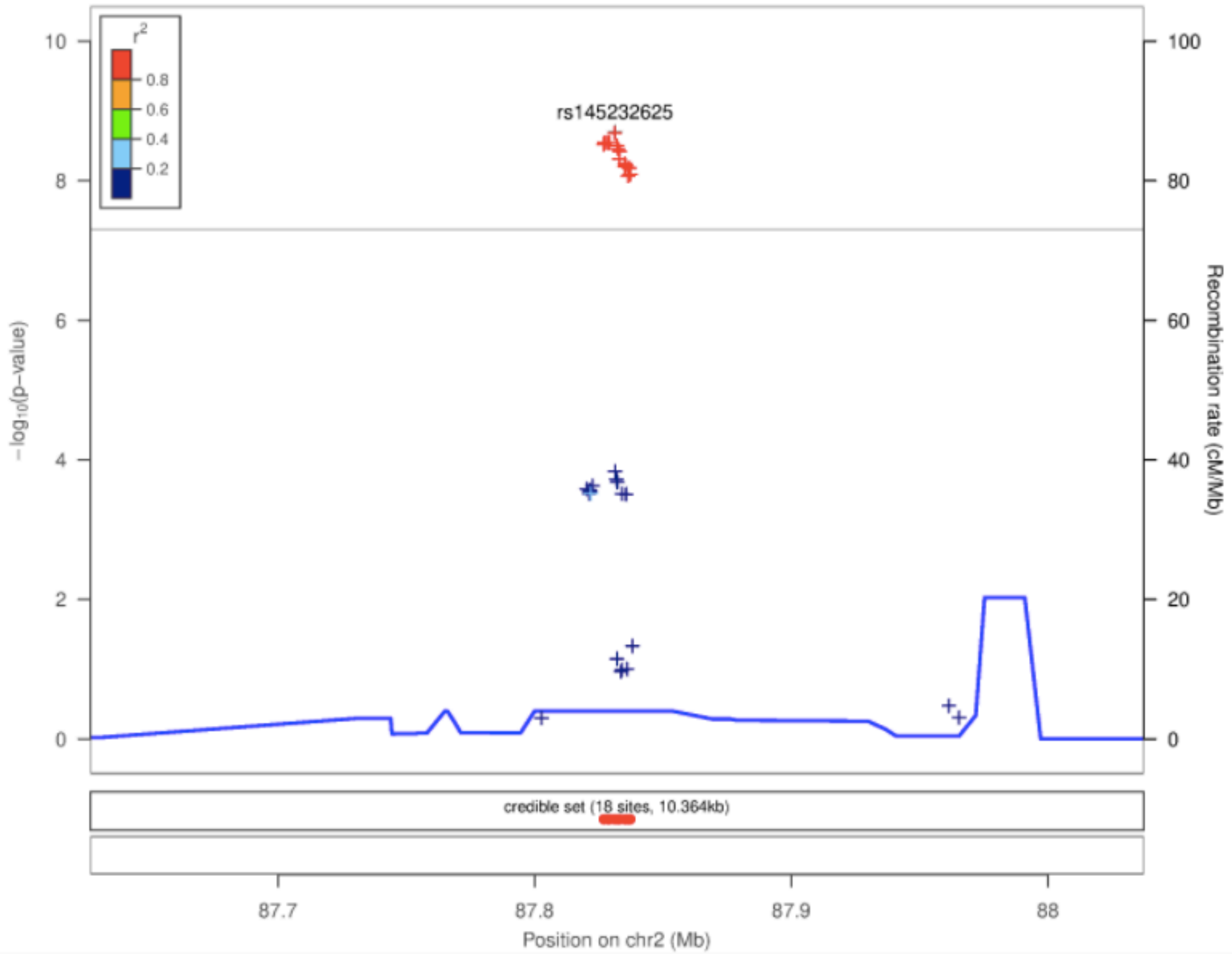

3.27 rs4933661: HECTD2–[]–PPP1R3C

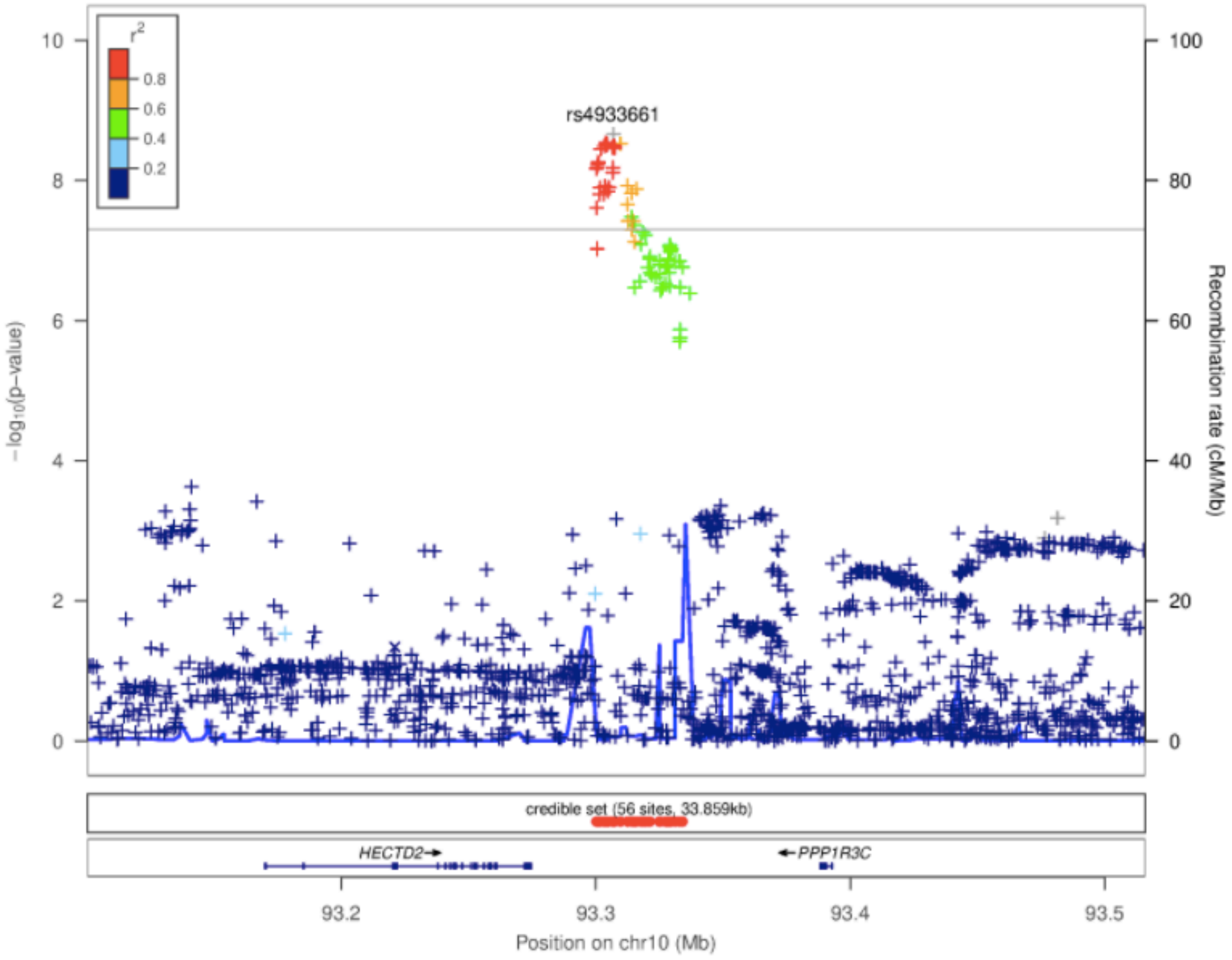

3.29 rs758049676: [CLEC16A]

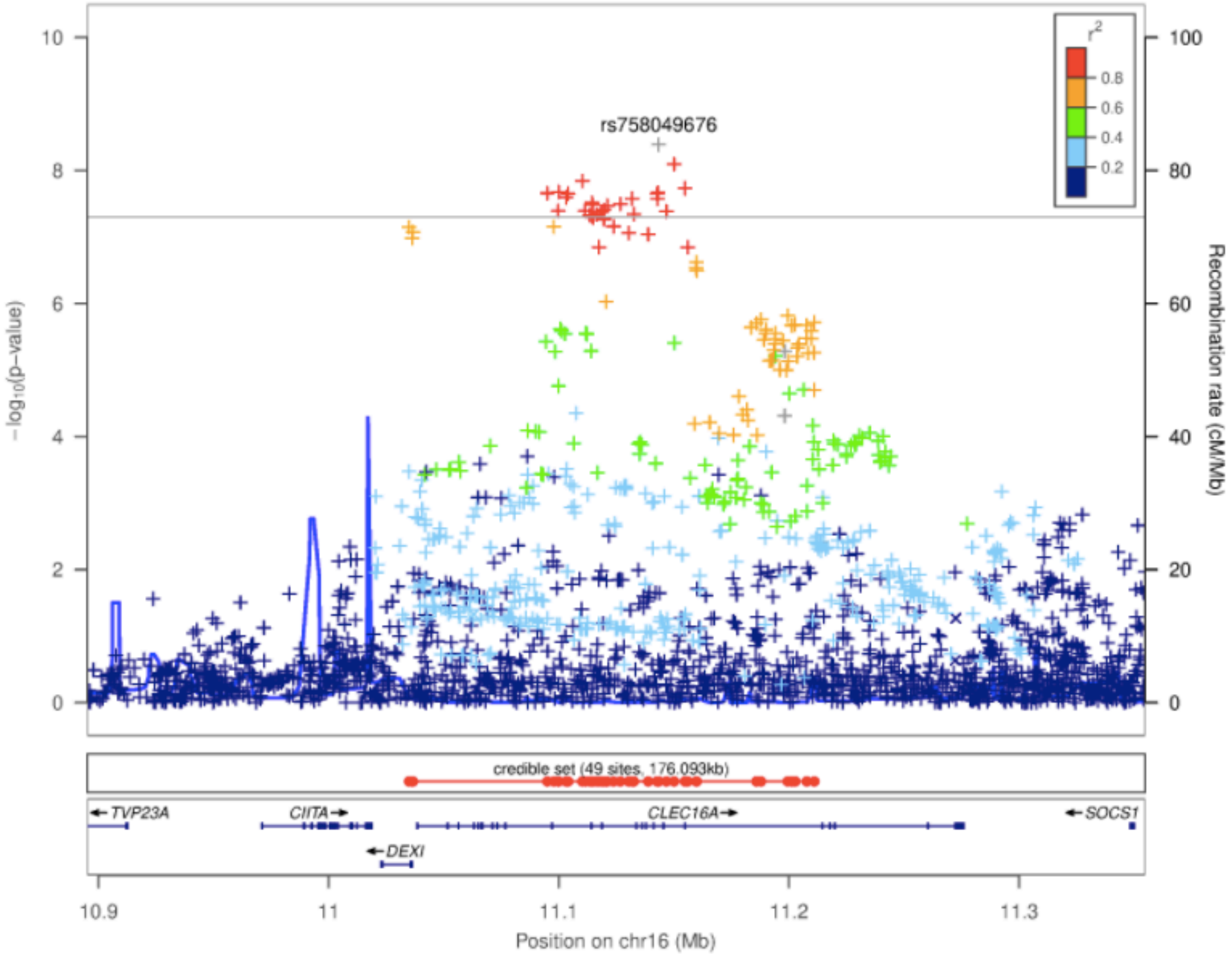

3.32 rs2286639: [SLC6A7]

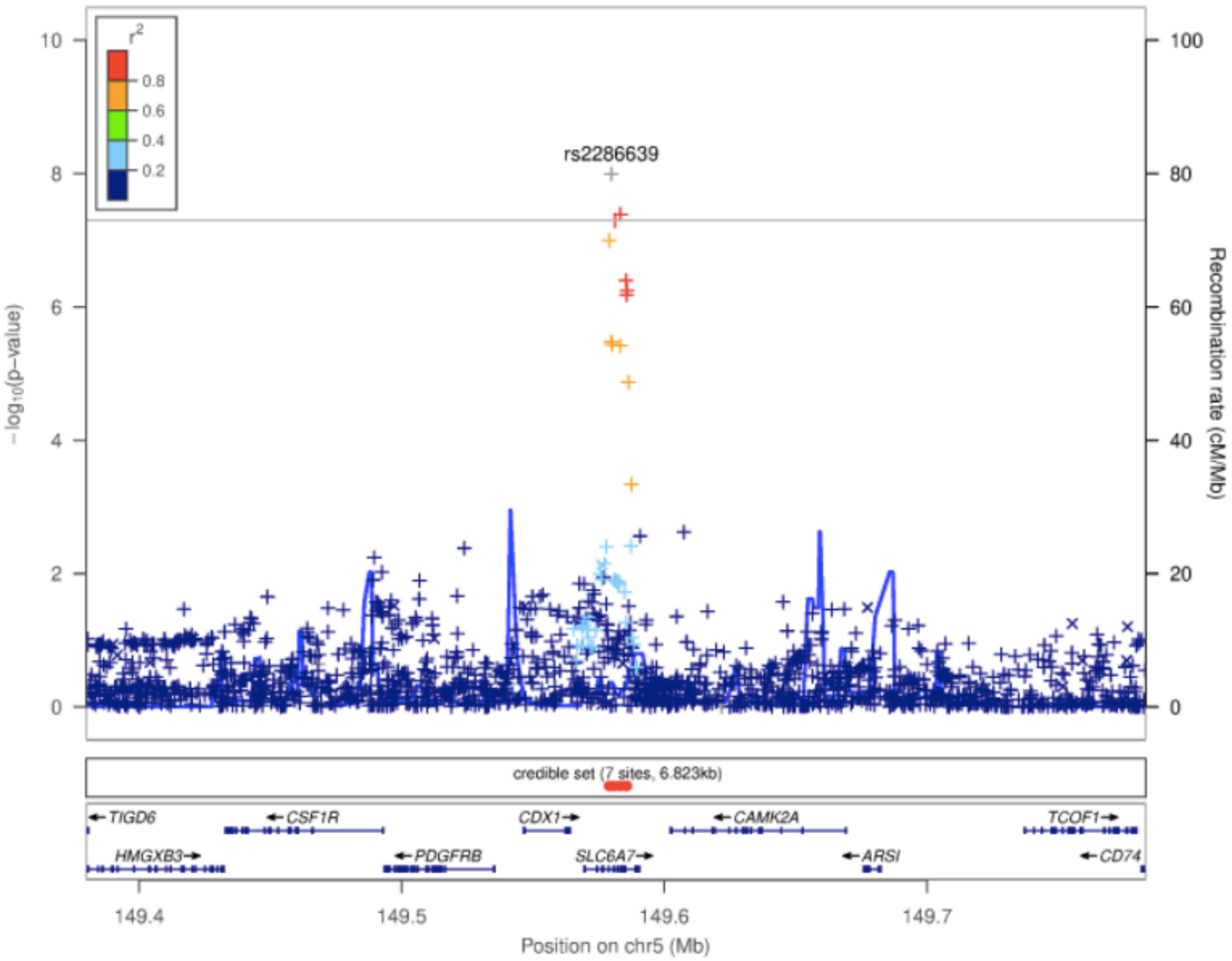

3.33 rs11064881: [CIT]

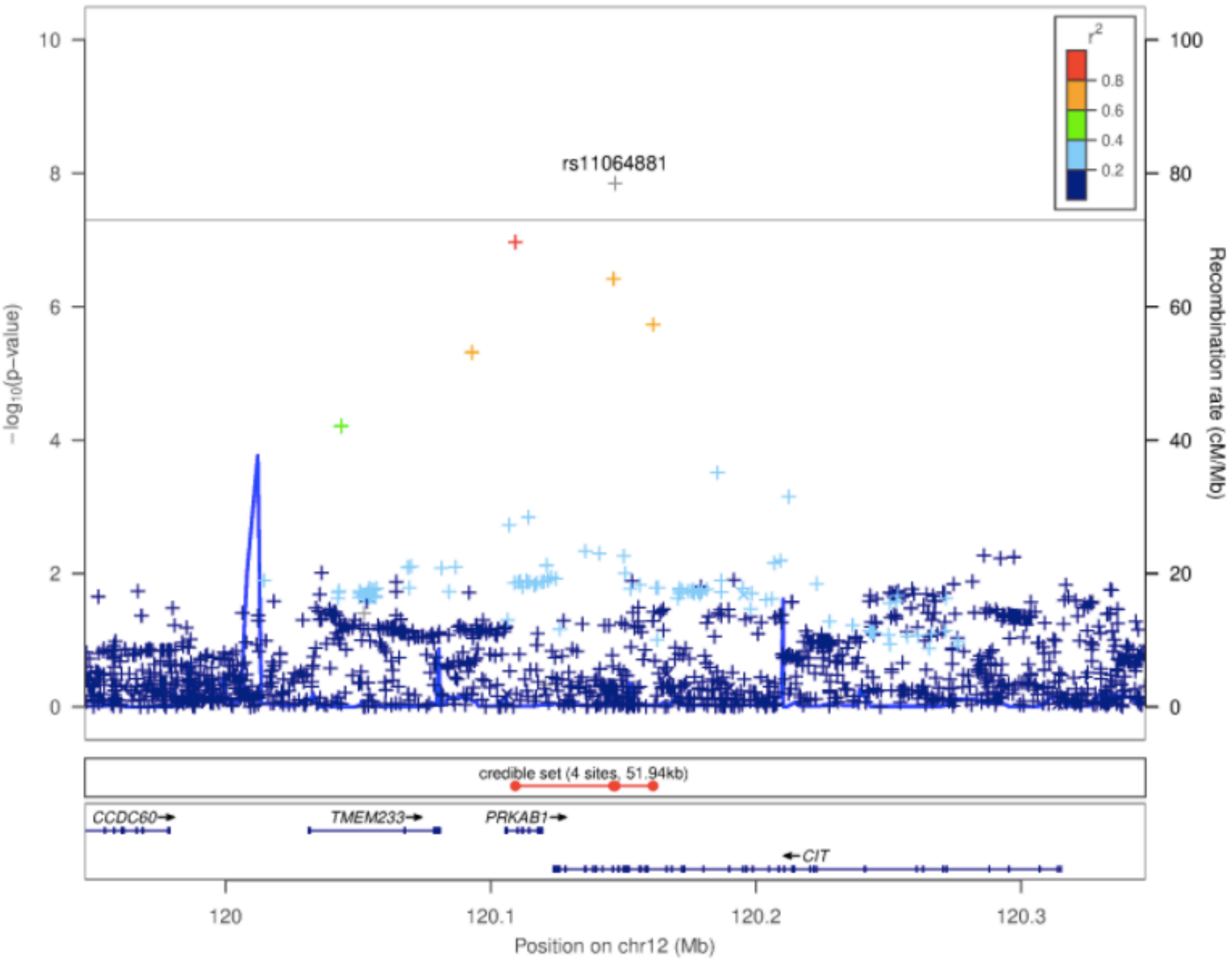

3.36 rs681343: [FUT2]

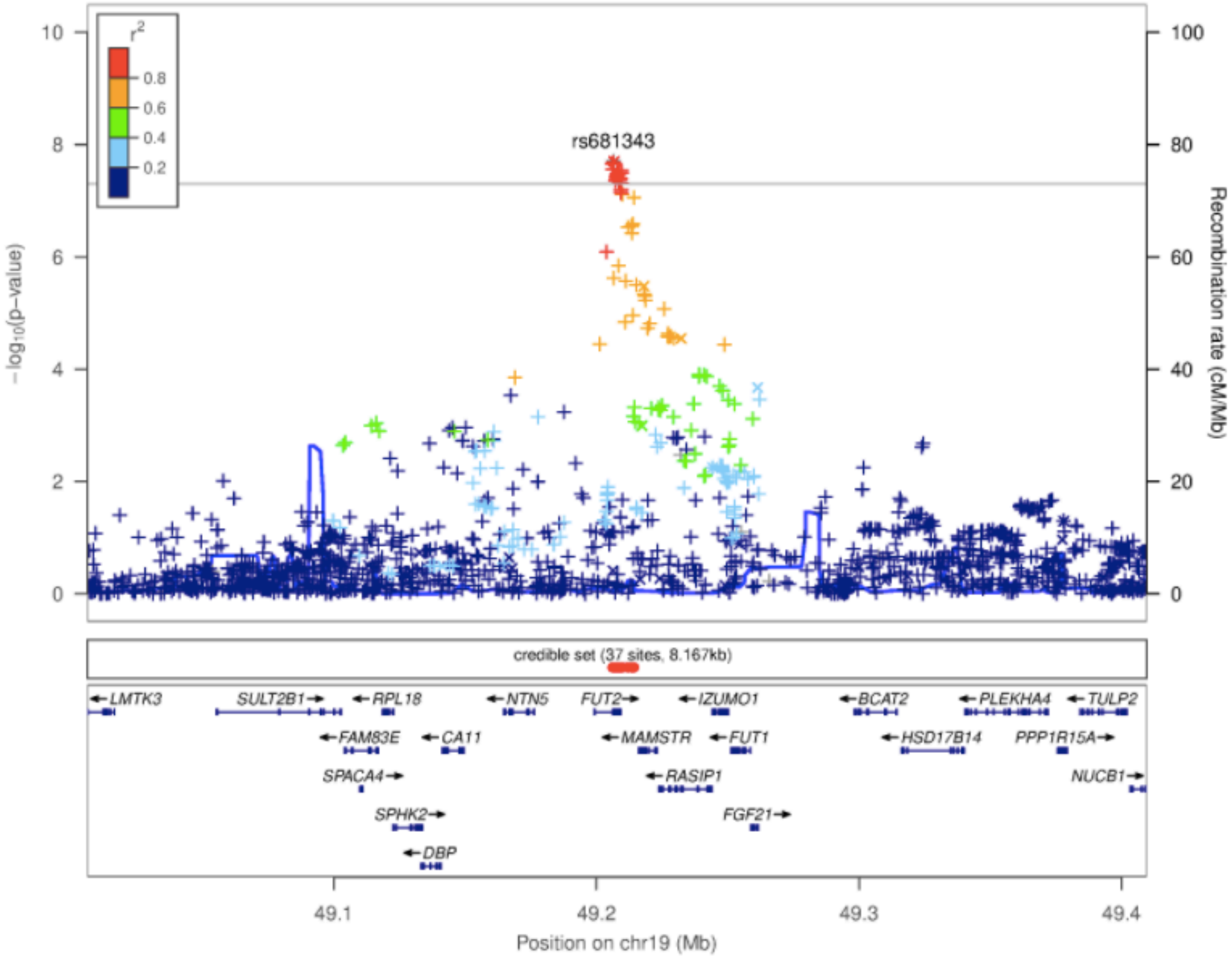

3.37 rs11679052: DYSF—[]–CYP26B1

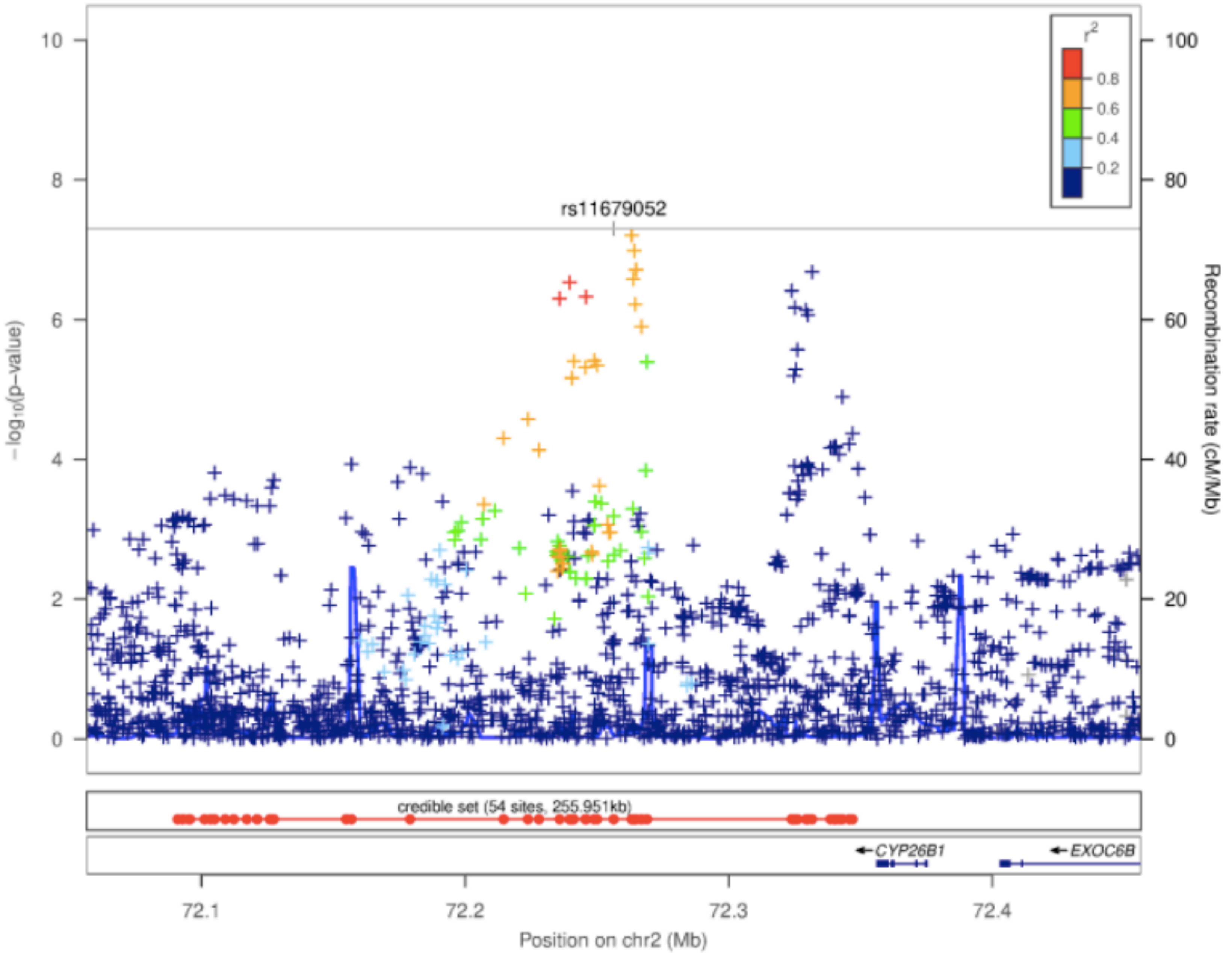

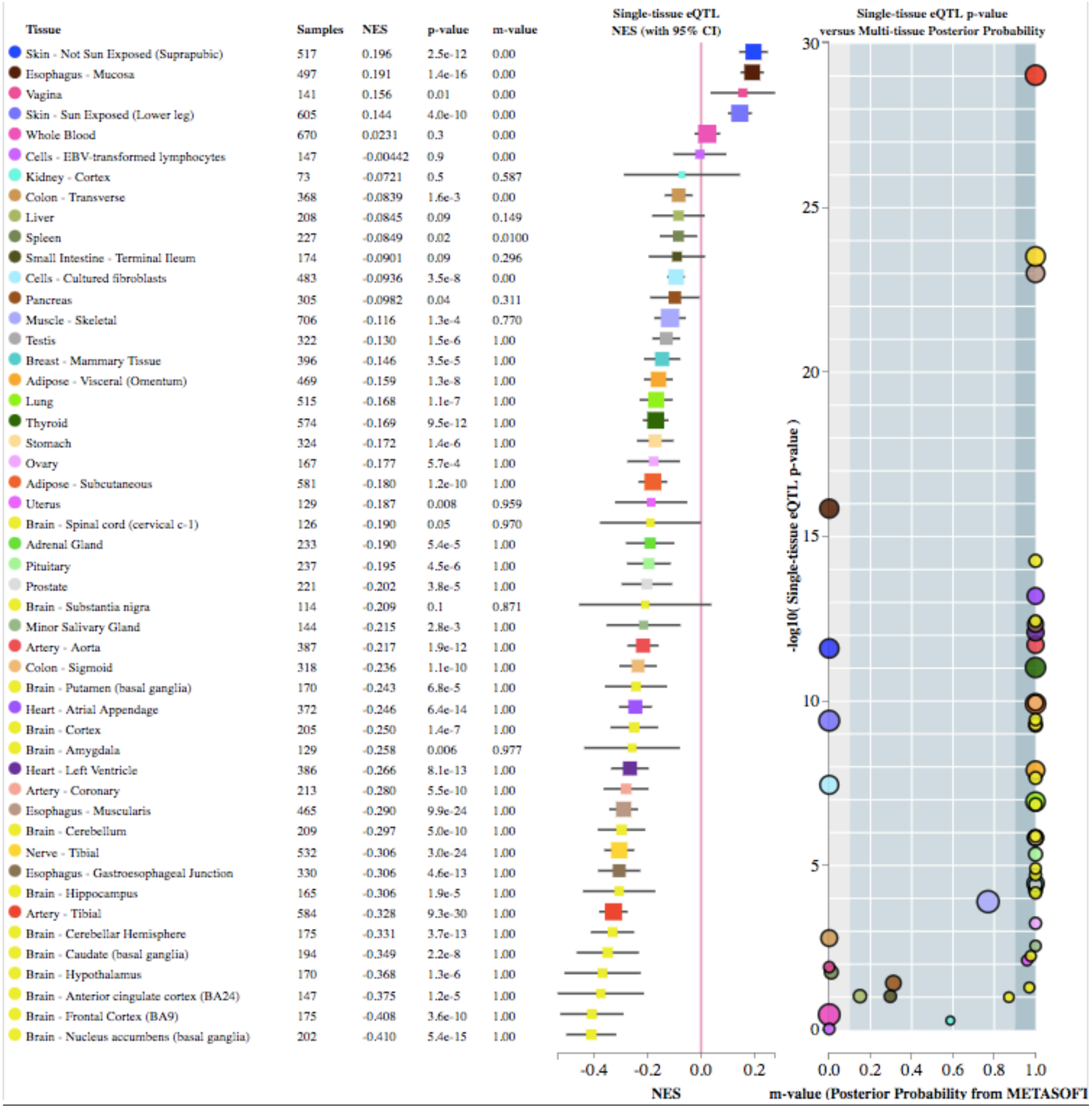

Fig. S5.

Multiple tissue eQTL plot of rs1049432 with TFAM expression. The data was downloaded from GTEx 30 October 2020. For most tissues evaluated, additional copies of the T allele are associated with decreased expression.

ID	Full	1st Third	2nd Third	3rd Third	NUMT474	NUMT228	Nearest Gene
rs1049432	1.57E-240	3.10E-204	5.48E-193	4.71E-263	2.33E-234	2.62E-236	TFAM
rs3843620	2.70E-58	4.51E-148	5.22E-70	**4.80E-02**	3.87E-60	1.56E-138	SIRT3
rs2519067	4.79E-50	9.49E-139	1.02E-64	**7.87E-01**	1.25E-52	2.74E-128	AL645608.2
rs1951197:C	7.81E-26	1.47E-116	2.01E-08	**2.71E-02**	1.98E-02	3.27E-58	AKAP6
rs28539606	7.67E-25	3.99E-18	2.05E-18	6.42E-33	2.85E-24	2.04E-21	HLA-DQB1
rs6471996	1.16E-21	**7.90E-01**	4.13E-158	**5.03E-01**	1.96E-24	1.72E-53	NKAIN3
rs4908650	6.45E-18	1.22E-18	2.86E-20	2.13E-11	1.41E-17	4.39E-19	CAMTA1
rs28602228	6.71E-17	4.97E-13	1.01E-11	2.15E-20	3.22E-16	2.92E-14	CSF2RA
rs4251979	9.51E-17	1.18E-13	3.79E-12	8.05E-20	1.22E-15	1.59E-15	IL1RN
rs139145400	1.37E-16	1.26E-13	1.63E-13	3.44E-19	1.48E-16	1.82E-16	PRDM8
rs1468326	2.72E-16	1.90E-14	7.99E-17	1.37E-13	6.82E-16	1.24E-16	WNK1
rs73081554	3.40E-15	3.53E-11	1.45E-10	1.39E-20	1.38E-14	4.47E-14	RPP14
rs571982832	4.23E-15	2.45E-10	3.95E-07	1.69E-27	1.81E-16	2.70E-06	FABP4
rs12461806	2.11E-14	1.71E-12	9.33E-11	3.25E-16	3.22E-14	1.36E-14	TINCR
rs74750282	5.46E-14	5.19E-49	1.67E-11	**8.91E-01**	7.67E-14	2.45E-34	MDFIC
rs370209610	8.26E-14	1.34E-11	7.95E-12	1.90E-14	1.02E-13	1.35E-12	IFNL4
rs58678340	4.68E-13	6.21E-12	1.35E-11	1.07E-11	6.80E-13	6.59E-13	TWNK/MRPL43
rs200605061	1.10E-12	4.53E-14	8.11E-11	1.37E-10	1.26E-12	6.16E-14	WDR72
rs7319964	1.17E-12	1.45E-11	4.56E-10	8.12E-13	2.71E-12	1.13E-12	KLF5
rs144047415	1.83E-12	5.85E-09	5.15E-12	1.28E-11	7.23E-13	6.62E-10	RAMP1
rs1469305	2.04E-12	5.97E-07	1.78E-08	1.21E-20	6.01E-12	5.89E-09	C15orf53
rs2149642	2.55E-12	1.88E-11	1.64E-10	4.74E-12	5.52E-12	1.01E-12	BMP2
rs143803034	3.06E-12	2.34E-07	1.93E-12	1.48E-12	4.80E-12	4.84E-10	CCZ1B
rs7728823	1.44E-10	6.58E-01	1.12E-49	1.74E-04	7.62E-12	6.42E-01	PCBD2
rs10063311	2.15E-10	1.71E-09	1.56E-09	8.68E-09	4.48E-10	5.12E-10	NDUFS4
rs145232625	4.81E-10	9.22E-06	7.76E-09	5.87E-12	3.37E-10	2.05E-08	PLGLB2
rs4933661	5.34E-10	2.68E-10	2.72E-12	1.58E-06	1.73E-09	1.06E-10	HECTD2
rs549950646	7.99E-10	4.22E-10	7.19E-08	1.17E-09	2.95E-10	8.37E-10	OR4A47
rs758049676	1.05E-09	2.38E-08	4.95E-07	7.02E-12	2.43E-09	3.22E-09	CLEC16A
rs1544065	1.20E-09	1.17E-09	3.40E-10	5.73E-07	1.45E-09	4.47E-10	ZNF618
rs35574789	2.68E-09	8.68E-08	4.07E-07	3.64E-10	3.14E-09	8.96E-09	CXCL6
rs2286639	2.78E-09	5.04E-07	4.28E-07	5.24E-12	1.90E-09	1.08E-07	SLC6A7
rs11064881	3.94E-09	1.09E-07	1.05E-07	2.84E-10	1.09E-08	1.55E-09	CIT/PRKAB1
rs191410813	4.52E-09	1.29E-08	8.60E-09	6.46E-08	1.10E-09	9.40E-08	PGLYRP2
rs645371:T	4.90E-09	1.59E-09	1.28E-08	7.33E-08	6.02E-09	3.08E-09	LONRF1
rs681343	5.62E-09	2.51E-07	2.26E-08	4.32E-08	2.50E-09	6.48E-08	FUT2/MAMSTR
rs11679052	1.54E-08	8.19E-08	1.49E-07	4.10E-09	2.25E-08	8.49E-08	CYP26B1

Table S1.

Evidence for NUMT interference with GWAS candidate loci. Loci are presented with the unadjusted *p* value for association using distinct quantifications of heteroplasmy. Full refers to

the use of all evaluated positions. The first (m.1-m.6425) second (m.6426-m.11947) and third (m.11947-m.16569) evaluations consider one-third of the mitochondrial positions in each analysis and have no overlap. NUMT 474 refers to the analysis in the absence of positions m.5583-6606 and NUMT 228 is evaluation in the absence of positions m.10270-15488. Positions removed from consideration because their association with heteroplasmy was region dependent are noted in red text.

Factor	Degrees of Freedom	Deviance	Residual Deviance	Heritability contribution
Null model	n.a.	n.a.	321853.41	n.a.
rs1049432	1	359.02	321494.39	0.112%
rs28539606	1	33.81	321460.57	0.011%
rs28602228	1	22.75	321437.82	0.007%
rs4251979	1	22.73	321415.09	0.007%
rs73081554	1	19.89	321395.20	0.006%
rs12461806	1	20.18	321375.02	0.006%
rs370209610	1	17.99	321357.04	0.006%
rs58678340	1	16.87	321340.16	0.005%
rs200605061	1	16.76	321323.41	0.005%
rs7319964	1	16.40	321307.01	0.005%
rs2149642	1	15.91	321291.10	0.005%
rs143803034	1	15.81	321275.30	0.005%
rs10063311	1	12.98	321262.31	0.004%
rs145232625	1	12.68	321249.63	0.004%
rs4933661	1	12.25	321237.38	0.004%
rs758049676	1	11.63	321225.75	0.004%
rs2286639	1	11.43	321214.32	0.004%
rs11064881	1	11.31	321203.01	0.004%
rs681343	1	11.31	321191.70	0.004%
rs11679052	1	10.59	321181.11	0.003%

Table S2.

The effect of the 20 loci were regressed in a linear fashion to determine the contribution of individual positions on the heritability of MtHz. Residuals of the original null model (log heteroplasmy ~ age + sex + principal components + log-autosomal variance) were regressed with adjusted $r^2 = 0.002069$.

	Haplogroup corrected		Haplogroup uncorrected	
SNP	p-value	effect	p-value	effect
rs1049432	5.4E-240	0.035	1.6E-240	0.035
rs28539606	1.2E-24	0.020	7.7E-25	0.020
rs28602228	6.0E-17	0.008	6.7E-17	0.008
rs4251979	1.2E-16	-0.008	9.5E-17	-0.008
rs73081554	1.5E-15	-0.014	3.4E-15	-0.014
rs12461806	1.6E-14	-0.012	2.1E-14	-0.012
rs370209610	7.6E-14	-0.023	8.3E-14	-0.023
rs200605061	4.3E-13	0.010	1.1E-12	0.009
rs58678340	6.8E-13	-0.025	4.7E-13	-0.026
rs2149642	1.1E-12	0.007	2.5E-12	0.007
rs7319964	1.5E-12	-0.006	1.2E-12	-0.006
rs143803034	4.8E-12	-0.015	3.1E-12	-0.015
rs145232625	2.3E-10	-0.006	4.8E-10	-0.006
rs10063311	2.6E-10	-0.006	2.2E-10	-0.006
rs4933661	6.7E-10	0.005	5.3E-10	0.005
rs758049676	1.0E-09	0.005	1.0E-09	0.005
rs681343	3.4E-09	0.005	5.6E-09	0.005
rs2286639	4.5E-09	-0.006	2.8E-09	-0.006
rs11064881	3.8E-09	-0.009	4.0E-09	-0.009
rs11679052	1.4E-08	-0.005	1.5E-08	-0.005

Table S3.

Haplogroup does not influence the association between MtHz and candidate SNPs. The top associated SNPs from Table 3 were re-evaluated with consideration of the impact of the 19 most common haplogroups. Corrected and uncorrected p-values for association with MtHz and effect size are shown. Significance values were not adjusted for genomic inflation.

Covariate	Estimate	Std. Error	t-value	Pr(>|t|)
age	-0.000104	0.000033	-3.2	0.0016
sexF	-0.032068	0.001167	-27.5	2.70E-166
pc.0	0.00391	0.000579	6.8	1.40E-11
pc.1	-0.000933	0.000582	-1.6	0.11
pc.2	0.00104	0.00058	1.8	0.073
pc.3	-0.000344	0.000582	-0.6	0.55
pc.4	0.001665	0.000584	2.8	0.0044
autosomal variance	0.65565	0.000885	740.6	<E-300
mtDNA copy number	0.265338	0.003963	67	<E-300

Table S4.

Effect of covariates on mean MtHz including age, sex, principal components, autosomal variance and relative mtDNA copy number on a logarithmic scale.

	Copy-number corrected		Uncorrected	
SNP	p-value	effect	p-value	effect
rs1049432	3.42E-231	0.034364	1.57E-240	0.035052
rs28539606	5.70E-20	0.017706	7.67E-25	0.019912
rs28602228	2.18E-12	0.006956	6.71E-17	0.008271
rs4251979	1.56E-14	-0.007128	9.51E-17	-0.007711
rs73081554	1.33E-12	-0.012681	3.40E-15	-0.014083
rs12461806	7.48E-14	-0.011468	2.11E-14	-0.011718
rs370209610	2.60E-12	-0.02117	8.26E-14	-0.022585
rs58678340	1.32E-12	-0.025059	4.68E-13	-0.025557
rs200605061	3.41E-13	0.009628	1.10E-12	0.009417
rs7319964	6.80E-12	-0.005679	1.17E-12	-0.005883
rs2149642	7.36E-13	0.007028	2.55E-12	0.006859
rs143803034	1.21E-10	-0.013852	3.06E-12	-0.015008
rs10063311	2.76E-10	-0.006245	2.15E-10	-0.006282
rs145232625	2.54E-09	-0.005508	4.81E-10	-0.005753
rs4933661	4.35E-11	0.005758	5.34E-10	0.005422
rs758049676	8.06E-09	0.004902	1.05E-09	0.005186
rs2286639	3.42E-08	-0.005634	2.78E-09	-0.006067
rs11064881	1.42E-08	-0.008908	3.94E-09	-0.009244
rs681343	4.87E-10	0.005108	5.62E-09	0.004783
rs11679052	9.59E-09	-0.004816	1.54E-08	-0.004748

Table S5.

mtDNA copy number does not influence the association between MtHz and candidate SNPs. The top associated SNPs from Table 3 were re-evaluated with consideration of mtDNA quantity evaluated by the LRR at mitochondrial positions. Corrected and uncorrected p-values for association with MtHz and effect size are shown. Significance values were not adjusted for genomic inflation.

Table S6.

PheWAS was performed by comparison with prior analyses. We evaluated the association of 19 SNPs associated with MtHz with 1123 traits. The table shows the SNP-phenotype interactions that met genome wide significance after correction ($0.05/1123/19 \sim 2.34 \times 10^{-6}$). The SNP, phenotype, number of cases and controls, association test *p* value, effect size (β), standard error and dosage of the minor allele in the unaffected (dose.b.0) and affected (dose.b.1) populations are shown. For quantitative traits, all individuals are listed as cases and the dose.b.1 is not provided.

assay.name	pheno	cases	controls	pvalue	effect	stderr	dose.b.0	dose.b.1
rs10063311	age_first_menses	390677	NA	8.28E-10	-0.01352154	0.002202399	0.449047755	NA
rs10063311	hair_curl	358411	NA	1.49E-25	0.03296623	0.003154983	0.446727171	NA
rs10063311	haircolor	497612	NA	8.65E-19	-0.02357194	0.002663042	0.447570339	NA
rs10063311	iqb.age_gray	378006	NA	1.02E-28	-0.08222283	0.007394595	0.447235694	NA
rs10063311	iqb.gray_amount	372689	NA	4.52E-66	0.077313196	0.004502232	0.44708915	NA
rs10063311	iqb.shampoo_type	587676	NA	7.09E-14	0.010587199	0.001414219	0.448503269	NA
rs10063311	unibrow	331791	NA	2.91E-10	-0.01511768	0.002398175	0.448780442	NA
rs1049432	pcos	61181	839824	5.64E-07	-0.03892159	0.007806838	0.366425643	0.354794433
rs11064881	chronotype	1619437	NA	9.24E-11	-0.0120804	0.001864564	1.853099923	NA
rs11064881	hashimotos	59604	1478372	2.90E-07	0.062311888	0.012239639	1.852729326	1.859797835
rs11064881	iqb.dandruff_frequency	949273	NA	2.33E-07	0.016583233	0.003207	1.853640682	NA
rs11064881	morning_person	629476	598088	8.94E-10	-0.03284959	0.005362014	1.855662676	1.851813439
rs11064881	severe_acne	169557	1401733	1.21E-09	-0.04457349	0.007300037	1.853370129	1.84784861
rs11679052	iqb.toenail_fungus	117094	399201	1.18E-10	-0.03212283	0.004984532	1.154887677	1.140398319
rs12461806	chronotype	1619437	NA	3.23E-10	0.011265847	0.001791861	1.826424094	NA
rs12461806	morning_person	629476	598088	2.27E-09	0.030703343	0.005136738	1.823944649	1.827992693
rs2149642	iqb.prone_to_bad_breath	216377	NA	6.46E-22	-0.03712941	0.003858513	1.550208438	NA
rs28539606	acne_as_teenager	422542	NA	2.86E-07	0.020164513	0.003928739	0.295000974	NA
rs28539606	actinic_keratosis	78578	1536429	1.34E-07	0.056007636	0.010573167	0.295588867	0.29603385
rs28539606	adult_onset_asthma	81619	853177	9.13E-32	-0.13692392	0.011785774	0.299695937	0.28061781
rs28539606	allergic_asthma	104543	861684	6.65E-80	-0.18813669	0.010064689	0.299725912	0.271284761
rs28539606	anemia	238175	1262531	2.11E-09	-0.03659738	0.006124385	0.296340813	0.288495482
rs28539606	ankylosing_spondylitis	13047	1543159	3.65E-08	0.126694413	0.022730656	0.294968467	0.312889261
rs28539606	any_allergy	648614	942179	7.12E-88	-0.08427213	0.004249466	0.30112458	0.28603535
rs28539606	any_animal_allergy	166363	974178	3.39E-135	-0.1824793	0.007480454	0.300941193	0.270530131
rs28539606	any_asthma	206177	823992	1.16E-125	-0.18537931	0.007849615	0.300118619	0.275316803
rs28539606	any_food_allergy	106711	1004984	8.75E-24	-0.08964811	0.008982646	0.300618392	0.284812951
rs28539606	any_non_melanoma_skin_can	197878	1382061	5.36E-31	0.081152669	0.006977163	0.295335555	0.298976876
rs28539606	any_plant_allergy	255833	948996	5.25E-60	-0.10029774	0.006178942	0.300922553	0.28167604
rs28539606	any_skin_cancer	239227	1369761	2.17E-25	0.067501772	0.006459686	0.295412399	0.297382228
rs28539606	asthma_efficacy_albuterol	68568	NA	2.01E-07	-0.05506133	0.010590827	0.27147738	NA
rs28539606	asthma_efficacy_short_beta_a	69955	NA	1.81E-07	-0.05457872	0.010460516	0.271803694	NA
rs28539606	basal_cell_carcinoma	155625	1455757	1.24E-40	0.101528103	0.007558439	0.295070806	0.301629974
rs28539606	blood_sugar_meds	68178	456532	1.21E-06	-0.05964032	0.012332674	0.295249608	0.284990952
rs28539606	cat_allergy	158067	1011379	6.56E-155	-0.19776863	0.007580679	0.300726979	0.267632839
rs28539606	celiac_HLA_all	17418	909817	1.70E-31	-0.34257819	0.030338101	0.18098716	0.152492747
rs28539606	cervical_cancer	12811	964141	4.80E-10	-0.14864522	0.024250568	0.29492056	0.266899095
rs28539606	cold_sores	233604	307800	2.14E-06	-0.0395045	0.008338398	0.297421775	0.291820611
rs28539606	crohns_or_ulcerative_colitis	40358	1588350	1.05E-19	0.125000751	0.013612123	0.29511966	0.315401302
rs28539606	crohns_or_ulcerative_colitis_v	54554	1573157	1.16E-19	0.106975184	0.011681026	0.295014626	0.312567671
rs28539606	current_asthma	49137	876625	1.68E-33	-0.17438943	0.014661534	0.29957015	0.273265132
rs28539606	dog_allergy	87809	1031313	5.59E-95	-0.20037438	0.009869573	0.300534218	0.266971497
rs28539606	dust_mites_allergy	171964	1010371	1.25E-54	-0.11190238	0.007250719	0.300603749	0.281046332
rs28539606	dust_mites_allergy_clean	165833	253006	7.49E-37	-0.11610265	0.009172663	0.298175606	0.28060909
rs28539606	eczema	195491	1401188	3.06E-15	0.050961721	0.006435782	0.294781807	0.303280242
rs28539606	eggs_allergy	12300	1052779	4.60E-07	-0.12944047	0.026006485	0.30024814	0.278173658
rs28539606	fish_allergy	10227	1053530	1.98E-10	-0.17593917	0.028151384	0.300232438	0.273150172
rs28539606	grasses_allergy	187557	1003033	9.49E-83	-0.1333331	0.006989914	0.30062937	0.276122569
rs28539606	graves	14455	1525548	3.14E-09	-0.13338752	0.022822463	0.295689965	0.275549852
rs28539606	hashimotos	59604	1478372	4.05E-76	-0.21401166	0.011827265	0.296470635	0.26641555
rs28539606	high_chol	192074	704263	1.03E-23	0.068143947	0.006762169	0.293534794	0.304486077
rs28539606	high_cholesterol_broad	629368	1349088	1.28E-13	0.032681932	0.004406853	0.29592425	0.298126472
rs28539606	high_ldl	126387	478790	1.70E-11	0.056457567	0.008361854	0.292803409	0.301354116
rs28539606	HIP_allergies	628721	923844	5.50E-89	-0.08467265	0.004242795	0.301189622	0.285978418
rs28539606	hyperthyroidism	26967	1539438	2.00E-06	-0.07963088	0.016891496	0.295666359	0.283176733
rs28539606	hypothyroidism	186956	1348462	2.87E-98	-0.14653859	0.007039673	0.297998629	0.27522677
rs28539606	iqb.childhood_ear_infections	214809	322672	1.30E-07	-0.04416927	0.008375562	0.29733381	0.290092975
rs28539606	iqb.dandruff_frequency	949273	NA	3.39E-34	0.038173602	0.003130673	0.295010453	NA
rs28539606	iqb.diagnosed_benign_migrat	9045	247927	2.89E-34	-0.34294985	0.029172443	0.295653122	0.239754855
rs28539606	iqb.mosquito_bite_size	498946	NA	5.67E-07	-0.02295636	0.004589231	0.294533113	NA
rs28539606	iqb.own_cat	335275	539165	5.32E-08	0.028489655	0.005232979	0.293498295	0.295402659

rs28539606	iqb.peanut_allergy_fh	24764	605951	4.28E-11	-0.10077961	0.015446377	0.295074399	0.275806429
rs28539606	iqb.pneumonia_frequency	396176	NA	8.81E-08	-0.02662569	0.004977109	0.293717795	NA
rs28539606	iqb.whooping_cough	45580	459379	2.79E-19	-0.13865314	0.015605981	0.297039959	0.278447241
rs28539606	juvenile_asthma	112441	855660	1.43E-105	-0.21524633	0.009998273	0.299828656	0.271009162
rs28539606	juvenile_asthma_not_current	32778	881682	4.58E-33	-0.20528682	0.017437549	0.299470681	0.270498799
rs28539606	juvenile_t1d	4440	1637886	4.84E-69	-0.7979413	0.049884656	0.296093908	0.189544078
rs28539606	kidney_stones	160784	1380904	1.64E-18	0.059560081	0.006752325	0.294397867	0.302421425
rs28539606	latex_allergy	39083	1044801	1.23E-07	-0.07496873	0.014275331	0.300335116	0.283801268
rs28539606	leukemia	4647	1614707	1.94E-06	-0.20208341	0.043346002	0.295639636	0.267641228
rs28539606	leukemia_type_CLL	2188	1532831	3.05E-07	-0.3112753	0.062836126	0.295221577	0.251961304
rs28539606	lichen_planus	9385	1625435	8.36E-49	0.372940318	0.02457351	0.295062293	0.361988809
rs28539606	metal_allergy	86097	372778	2.91E-07	-0.05479334	0.010709468	0.292729522	0.283482697
rs28539606	milk_allergy	21790	1050137	7.81E-09	-0.11037514	0.019329512	0.300236022	0.280678091
rs28539606	mold_allergy	166710	1009336	5.69E-48	-0.10607708	0.007348952	0.300550238	0.280519297
rs28539606	no_allergies	969101	744269	7.18E-62	0.067046258	0.004045642	0.288079052	0.300652938
rs28539606	non_hodgkins_lymphoma_dx_	15037	1602464	5.25E-09	0.130862554	0.022143962	0.295384073	0.316259432
rs28539606	other_animal_allergy	40280	1044775	1.22E-55	-0.22020837	0.014320085	0.300312874	0.263488649
rs28539606	other_food_allergy	61878	1038378	7.96E-10	-0.06758403	0.011064287	0.300310203	0.287666256
rs28539606	other_plant_allergy	61869	1038270	6.65E-11	-0.07223893	0.011139227	0.300336586	0.28490033
rs28539606	peanut_allergy	11450	1053573	7.55E-07	-0.1275076	0.026115319	0.300217752	0.278791857
rs28539606	peanut_allergy_broad	12128	1078943	1.00E-07	-0.13399412	0.02550586	0.300222064	0.277861761
rs28539606	pediatric_ibd	5976	1602534	4.87E-07	0.173492736	0.033952655	0.295134548	0.325465949
rs28539606	plantar_warts	141186	219242	2.25E-06	-0.04698396	0.009945214	0.296624666	0.288827198
rs28539606	pollen_allergy	254868	983756	1.62E-65	-0.10361173	0.006103199	0.300741448	0.280731837
rs28539606	positive_tb_test	34228	531918	4.35E-12	-0.12144193	0.017712799	0.295602182	0.282736983
rs28539606	rhinitis	277227	953957	3.09E-74	-0.10915657	0.006029672	0.300989339	0.280899065
rs28539606	rosacea_all	131418	1511387	1.29E-29	0.087324592	0.007673784	0.294686479	0.305138582
rs28539606	rosacea_severity	251085	NA	7.71E-07	0.108765157	0.022005719	0.294132802	NA
rs28539606	rosacea_w_treatment	28689	1502486	5.49E-08	0.081154158	0.014824026	0.294828612	0.303052953
rs28539606	seasonal_allergies_broad	297070	971775	6.45E-64	-0.09711361	0.005788429	0.300812857	0.282089036
rs28539606	severe_acne	169557	1401733	3.47E-07	0.034458925	0.006744572	0.295114107	0.300243169
rs28539606	severe_asthma	5674	885481	1.98E-08	-0.32679163	0.059341384	0.299463847	0.268959088
rs28539606	severe_asthma_new_experim	15584	882858	1.28E-13	-0.1865517	0.025608226	0.299498314	0.271147654
rs28539606	squamous_cell_carcinoma	82062	1532976	1.86E-06	0.04871323	0.010178612	0.295689276	0.294086146
rs28539606	steroid_meds	260865	336193	2.24E-06	-0.04134486	0.008742166	0.299214774	0.290250225
rs28539606	t1d	12103	1634497	6.03E-93	-0.55616013	0.028912826	0.296141325	0.218719249
rs28539606	took_meds_anti_tnf_alpha	23073	1635056	2.78E-11	0.119950057	0.017818661	0.295106382	0.312071009
rs28539606	tree_nuts_allergy	19641	1050517	6.79E-17	-0.16619036	0.020238842	0.300321937	0.273338676
rs28539606	trees_allergy	181014	1004556	1.23E-44	-0.09840175	0.00707285	0.30055607	0.281513426
rs28539606	ulcerative_colitis	27148	1603796	1.54E-29	0.185602727	0.016176271	0.295109513	0.325165179
rs28539606	urinary_tract_inf	124609	1358335	1.76E-07	0.039892724	0.007614469	0.294813861	0.298092189
rs28539606	weeds_allergy	192578	1001777	9.85E-56	-0.10738257	0.006883065	0.300588165	0.279618957
rs28539606	wheat_allergy	17838	1051118	5.43E-10	-0.1342254	0.021913912	0.30023869	0.276970105
rs4251979	acne_as_teenager	422542	NA	9.17E-08	-0.01214025	0.002272373	1.463673856	NA
rs4251979	kidney_stones	160784	1380904	1.85E-17	-0.03632218	0.004263173	1.465956533	1.451794946
rs4251979	severe_acne	169557	1401733	1.64E-12	-0.02953723	0.004175788	1.465554604	1.454211306
rs4251979	weight_qnorm	1689148	NA	2.32E-06	-0.00583657	0.001235574	1.464791787	NA
rs4933661	bmi_qnorm	1639524	NA	2.16E-06	0.005512241	0.001163467	0.713077448	NA
rs4933661	height	1689909	NA	1.64E-06	-0.01467396	0.003060979	0.713061888	NA
rs4933661	urinary_tract_inf	124609	1358335	5.40E-07	-0.02303204	0.004599637	0.713501452	0.703667048
rs4933661	vaginal_inf	38935	851776	5.71E-09	0.045383434	0.007777047	0.710351975	0.729779698
rs4933661	yeast_infections	345141	NA	4.45E-31	0.031820247	0.002744468	0.708180762	NA
rs681343	anemia	238175	1262531	1.18E-23	0.03334549	0.003326048	0.965358409	0.986341353
rs681343	any_cvd	563775	1080042	2.38E-10	0.016496325	0.002604138	0.964246542	0.97448385
rs681343	basal_cell_carcinoma	155625	1455757	1.16E-06	-0.01961332	0.004034549	0.966901172	0.97585481
rs681343	bladder_cancer_dx_or_fh	18095	1651577	3.41E-12	-0.0745315	0.010716656	0.968081184	0.931970732
rs681343	bladder_cancer_fh	12313	398422	8.12E-08	-0.07033367	0.013119672	0.969545518	0.934934065
rs681343	blood_clots	55282	1571038	4.57E-08	0.033878702	0.006196268	0.967356996	0.9861764
rs681343	bmi	1639771	NA	1.87E-12	-0.04567854	0.006485105	0.967884416	NA
rs681343	bmi_qnorm	1639524	NA	6.54E-15	-0.00856162	0.001098619	0.967895396	NA
rs681343	body_type	150069	288327	2.11E-08	-0.0282838	0.005048333	0.976936996	0.961643419
rs681343	cavities_many	303738	96113	8.41E-10	0.035424625	0.005773112	0.959596296	0.975200714

rs681343	chronic_constipation	57697	633137	1.03E-06	0.030591821	0.006261617	0.968380326	0.989266327
rs681343	chronotype	1619437	NA	4.29E-25	-0.00958722	0.000926493	0.968077851	NA
rs681343	cold_sores	233604	307800	3.21E-08	0.021835237	0.003948842	0.965733725	0.976824033
rs681343	cold_vs_hot	138740	153365	1.30E-06	0.026735055	0.005524038	0.965483926	0.97384837
rs681343	colds_last_year	579912	NA	1.10E-64	-0.02069637	0.001218516	0.970950926	NA
rs681343	colon_polyps	269006	1284532	1.69E-13	0.024075176	0.003266082	0.96555907	0.98159871
rs681343	crohns	17023	1613571	9.25E-16	0.088024148	0.010953254	0.96755933	1.009707973
rs681343	crohns_or_ulcerative_colitis	40358	1588350	1.99E-08	0.040262465	0.007172784	0.967481846	0.986557384
rs681343	crohns_or_ulcerative_colitis_v	54554	1573157	6.83E-07	0.030813118	0.006203906	0.967437825	0.9818113
rs681343	crohns_w_fistula_or_stricture	4758	1618267	5.59E-07	0.103218714	0.020625652	0.967621445	1.016705816
rs681343	deep_vein_thrombosis	32216	1637272	3.45E-08	0.044350573	0.008038598	0.967487126	0.990319883
rs681343	deep_vein_thrombosis_w_prc	48840	1564319	8.68E-11	0.042664144	0.006575077	0.967286781	0.990879464
rs681343	gallstones_or_gall_bladder_re	72915	544299	2.74E-26	0.061342453	0.005783139	0.966776429	1.003231596
rs681343	hashimotos	59604	1478372	1.10E-13	0.044622121	0.00600639	0.967503954	0.98956174
rs681343	healthy_old	116163	1084694	2.28E-28	-0.04862247	0.004403015	0.97467368	0.950643644
rs681343	heart_metabolic_disease_60	527510	162245	6.63E-38	0.052796573	0.004103497	0.951936842	0.975998225
rs681343	heart_metabolic_disease_70	284187	56447	2.01E-12	0.046369825	0.006594243	0.951067517	0.971805703
rs681343	heart_metabolic_disease_burc	1611466	NA	9.56E-24	0.010822014	0.001077231	0.968293188	NA
rs681343	height	1689909	NA	1.04E-15	-0.0231893	0.00289049	0.9678623	NA
rs681343	height_qnorm	1689296	NA	2.72E-16	-0.0088882	0.001085863	0.967900057	NA
rs681343	hemorrhoids	232143	343553	8.20E-07	0.019600162	0.003975244	0.966462561	0.977067751
rs681343	high_blood_pressure	493911	1131414	1.48E-11	0.018301951	0.002711568	0.964608492	0.975690414
rs681343	high_chol	192074	704263	8.48E-101	0.078504294	0.003683909	0.962121071	0.999884722
rs681343	high_cholesterol_broad	629368	1349088	2.12E-100	0.050426414	0.002370887	0.959688112	0.984595366
rs681343	high_ldl	126387	478790	7.40E-31	0.052159536	0.004516119	0.962535356	0.988458606
rs681343	high_tg	116217	363466	2.84E-25	0.050337613	0.004846275	0.963467061	0.991642484
rs681343	HIP_anemia_cause_deficiency	136240	1378915	1.45E-13	0.030647572	0.004145814	0.966731054	0.987220122
rs681343	hp_alcohol_2wks	1588481	NA	4.92E-32	0.021911748	0.001859964	0.967928855	NA
rs681343	hp_diet_quality	1552459	NA	2.40E-08	0.008790457	0.001575188	0.968229436	NA
rs681343	hypothyroidism	186956	1348462	1.69E-10	0.023378608	0.003660075	0.96640235	0.980604754
rs681343	iqb.bedwetting_frequency	547585	NA	1.54E-06	-0.01020391	0.00212304	0.970396053	NA
rs681343	iqb.childhood_ear_infections	214809	322672	1.51E-143	-0.10171627	0.003990249	0.988257038	0.94031676
rs681343	iqb.chocolate_frequency	354646	NA	4.22E-23	-0.0189353	0.001912811	0.970852278	NA
rs681343	iqb.dry_skin_frequency	531135	NA	4.64E-11	0.013802481	0.002097022	0.971621416	NA
rs681343	iqb.fresh_cantaloupe_yummy	342970	71887	2.26E-06	0.028159699	0.005955745	0.966042549	0.973577132
rs681343	iqb.gallstones	72043	535026	1.63E-26	0.061979635	0.005816563	0.966854699	1.003269571
rs681343	iqb.influenza_last_12mo	124771	502056	4.58E-17	-0.03796775	0.004522565	0.972253641	0.952122144
rs681343	iqb.licorice	186346	226828	1.28E-22	0.045125651	0.004611505	0.965073685	0.981105816
rs681343	iqb.liking_sweets	338025	NA	4.16E-61	0.050446089	0.003058187	0.971530048	NA
rs681343	iqb.low_hdl	117106	426358	2.07E-07	0.025035754	0.004821398	0.967574774	0.981479428
rs681343	iqb.norovirus_ever	140051	334786	0	-0.18940865	0.004602112	0.993444759	0.905728257
rs681343	iqb.pastry_frequency	378933	NA	1.35E-16	0.021599295	0.002611914	0.971688145	NA
rs681343	iqb.spit_frequency	360235	NA	1.55E-83	-0.1063459	0.005490603	0.971783448	NA
rs681343	iqb.whooping_cough	45580	459379	4.92E-09	0.041708982	0.007129505	0.96811482	0.992368093
rs681343	juvenile_asthma	112441	855660	2.05E-09	-0.02763488	0.004611323	0.968170675	0.964054886
rs681343	juvenile_t1d	4440	1637886	3.83E-07	0.108459355	0.02136694	0.967762233	1.025588235
rs681343	kidney_stones	160784	1380904	1.79E-15	0.03010717	0.003784461	0.966520384	0.984956173
rs681343	measles	291667	391515	1.76E-14	0.040717993	0.005311241	0.963711885	0.976954147
rs681343	morning_person	629476	598088	1.42E-24	-0.02732676	0.002670683	0.975008598	0.961749945
rs681343	motionsick_combined	424001	NA	1.68E-06	-0.00942894	0.0019689	0.970255427	NA
rs681343	mumps	194400	341700	0	0.227220278	0.005241474	0.944914542	1.022451279
rs681343	number_of_cavities	400874	NA	4.80E-08	0.006447697	0.001181192	0.971617741	NA
rs681343	pancreatitis	19603	1438859	1.59E-10	0.065519289	0.010242228	0.967834495	1.000531786
rs681343	pediatric_ibd	5976	1602534	4.79E-07	0.092799322	0.018432426	0.967614857	1.008159435
rs681343	psoriasis	87077	1520979	4.40E-21	0.046756602	0.004961912	0.966756529	0.993887422
rs681343	psoriasis_plaque	11319	1551983	7.38E-07	0.066404211	0.013411846	0.966973085	1.005789494
rs681343	psoriasis_plaque_severe	9163	1605675	2.63E-09	0.088666722	0.014895699	0.966821034	1.019644221
rs681343	red_meat_servings	1598641	NA	1.32E-34	-0.01427441	0.001163382	0.968117875	NA
rs681343	rosacea_severity	251085	NA	2.28E-06	0.052599658	0.011128703	0.970071333	NA
rs681343	salty_tooth	259764	457263	1.04E-109	-0.07788799	0.003501763	0.982283029	0.946337604
rs681343	stroke_w_proxy	32946	1602476	5.65E-08	0.043341022	0.007982183	0.967420221	0.994753069
rs681343	sweet_tooth	184479	533386	2.42E-56	0.060901199	0.003851021	0.962992781	0.99323135

rs681343	sweet_v_salty	175626	251096	1.13E-113	0.100678716	0.004445593	0.945881969	0.993776599
rs681343	t1d	12103	1634497	4.50E-10	0.080874929	0.012970167	0.967731368	1.012100387
rs681343	took_meds_anti_tnf_alpha	23073	1635056	1.80E-07	0.049212148	0.009428364	0.967463558	0.995398888
rs681343	ulcer	73643	1431667	1.36E-08	0.030693157	0.005404586	0.967547483	0.982572564
rs681343	vaginal_inf	38935	851776	5.89E-11	0.048310107	0.007379079	0.968165131	0.994481904
rs681343	vegetable_servings	1562427	NA	7.55E-08	-0.00490863	0.000912811	0.96829513	NA
rs681343	weight	1689226	NA	1.49E-21	-0.41227105	0.043233437	0.967876322	NA
rs681343	weight_qnorm	1689148	NA	8.27E-27	-0.01161723	0.001083756	0.967877907	NA
rs681343	yeast_infections	345141	NA	1.20E-24	0.026537897	0.002589221	0.972832439	NA
rs73081554	chronotype	1619437	NA	4.12E-10	-0.01202634	0.001924365	0.135253231	NA
rs73081554	hashimotos	59604	1478372	8.54E-08	0.066322921	0.01228819	0.134925474	0.142497126
rs73081554	hypothyroidism	186956	1348462	1.16E-07	0.040128987	0.007545502	0.134400177	0.140104191
rs73081554	morning_person	629476	598088	1.22E-11	-0.03768984	0.005560802	0.137589416	0.133174608
rs7319964	any_allergy	648614	942179	1.88E-12	0.016426376	0.002332312	1.075128203	1.082857422
rs7319964	any_animal_allergy	166363	974178	3.15E-13	0.028215366	0.003872725	1.07526706	1.088686296
rs7319964	any_plant_allergy	255833	948996	1.93E-09	0.019541916	0.00325534	1.07524483	1.08488701
rs7319964	bmi	1639771	NA	1.07E-06	-0.03191446	0.00654294	1.07812897	NA
rs7319964	bmi_qnorm	1639524	NA	1.69E-06	-0.00533766	0.001114882	1.078123123	NA
rs7319964	cat_allergy	158067	1011379	2.62E-13	0.02882427	0.003942826	1.075026797	1.088838299
rs7319964	chronotype	1619437	NA	1.13E-08	0.005338029	0.000934928	1.077712084	NA
rs7319964	dog_allergy	87809	1031313	1.96E-10	0.032511668	0.005110531	1.075160341	1.090683852
rs7319964	dust_mites_allergy	171964	1010371	9.74E-08	0.020367738	0.003821042	1.075333303	1.085044808
rs7319964	grasses_allergy	187557	1003033	2.28E-10	0.023259817	0.003668916	1.07513432	1.086451403
rs7319964	HIP_allergies	628721	923844	8.84E-12	0.016121306	0.002362481	1.075147891	1.082726651
rs7319964	iqb.nose_bleeds	30837	396423	1.44E-06	0.041029022	0.008517466	1.078174603	1.098008313
rs7319964	morning_person	629476	598088	1.74E-10	0.017197806	0.002694573	1.07346136	1.082626009
rs7319964	no_allergies	969101	744269	4.20E-09	-0.01316312	0.002240106	1.08135217	1.075096168
rs7319964	other_animal_allergy	40280	1044775	2.07E-08	0.041154119	0.007345591	1.075367019	1.094853105
rs7319964	pollen_allergy	254868	983756	4.58E-10	0.02023951	0.003247865	1.075072349	1.085054781
rs7319964	rhinitis	277227	953957	9.84E-12	0.021514591	0.003160383	1.075233867	1.085579742
rs7319964	seasonal_allergies_broad	297070	971775	1.80E-08	0.017361049	0.003083761	1.075116037	1.08379462
rs7319964	trees_allergy	181014	1004556	1.41E-06	0.017974071	0.003726623	1.075107315	1.083972777
rs7319964	weeds_allergy	192578	1001777	2.45E-10	0.022948805	0.003626095	1.075215916	1.086447361
rs758049676	acne_as_teenager	422542	NA	1.36E-124	-0.04953033	0.00208561	0.857400748	NA
rs758049676	allergic_asthma	104543	861684	2.00E-21	-0.04678215	0.004926205	0.865842507	0.842372298
rs758049676	any_allergy	648614	942179	3.46E-78	-0.0450242	0.002405965	0.870778648	0.850436573
rs758049676	any_animal_allergy	166363	974178	1.50E-52	-0.06099634	0.004002421	0.871546999	0.842509886
rs758049676	any_asthma	206177	823992	5.19E-31	-0.04252769	0.003674788	0.865932223	0.846086135
rs758049676	any_drug_allergy	133412	994232	9.99E-43	-0.06062744	0.004429872	0.871706235	0.842041373
rs758049676	any_food_allergy	106711	1004984	1.13E-14	-0.0373281	0.004836354	0.871136971	0.853179606
rs758049676	any_plant_allergy	255833	948996	3.40E-41	-0.04516356	0.003361861	0.871712735	0.847895419
rs758049676	back_hair	350599	NA	3.41E-08	0.015166186	0.00274789	0.863335774	NA
rs758049676	cat_allergy	158067	1011379	8.08E-57	-0.06467312	0.004076211	0.871573643	0.840958283
rs758049676	chronic_hives	137804	1404307	2.76E-13	-0.03095667	0.004239741	0.86436357	0.85003923
rs758049676	current_asthma	49137	876625	1.40E-10	-0.04441064	0.006928982	0.865644429	0.842405792
rs758049676	dog_allergy	87809	1031313	9.18E-39	-0.068723	0.005284992	0.871585071	0.839041319
rs758049676	dust_mites_allergy	171964	1010371	4.06E-38	-0.05089922	0.0039466	0.871746207	0.848068333
rs758049676	dust_mites_allergy_clean	165833	253006	6.75E-19	-0.04230099	0.0047656	0.865171897	0.848232191
rs758049676	eczema	195491	1401188	1.18E-13	-0.02665315	0.003594373	0.864464199	0.854508331
rs758049676	grasses_allergy	187557	1003033	2.00E-41	-0.05104952	0.003789804	0.871691478	0.845519749
rs758049676	HIP_allergies	628721	923844	1.28E-69	-0.04297781	0.002437506	0.870136848	0.850858889
rs758049676	insect_allergy	48437	1042831	3.75E-07	-0.03525767	0.006943837	0.871467141	0.852334785
rs758049676	iqb.bruise_easily	200257	284323	9.24E-10	0.02852157	0.004658792	0.856956685	0.866459001
rs758049676	iqb.childhood_ear_infections	214809	322672	1.23E-06	-0.02003729	0.004131361	0.866020233	0.854764506
rs758049676	iqb.gray_amount	372689	NA	2.18E-07	-0.02012872	0.00388368	0.860587148	NA
rs758049676	iqb.shampoo_type	587676	NA	1.67E-24	0.012455631	0.001219118	0.861171297	NA
rs758049676	iqb.stretch_marks	285414	404710	6.19E-18	-0.03449146	0.003997774	0.868250791	0.852715
rs758049676	iqb.toenail_fungus	117094	399201	1.89E-06	-0.02421003	0.005082129	0.862820075	0.852097883
rs758049676	juvenile_asthma	112441	855660	3.64E-33	-0.05756209	0.004802798	0.865851301	0.839947983
rs758049676	juvenile_asthma_not_current	32778	881682	1.23E-10	-0.05395572	0.008393608	0.865729074	0.840188391
rs758049676	latex_allergy	39083	1044801	4.28E-10	-0.04825401	0.007736899	0.871428191	0.844704899
rs758049676	metal_allergy	86097	372778	6.35E-09	-0.03459229	0.005959157	0.860469456	0.844038049

rs758049676	milk_allergy	21790	1050137	2.11E-08	-0.05709195	0.010203892	0.87131584	0.843683647
rs758049676	mold_allergy	166710	1009336	1.84E-31	-0.04663006	0.003999176	0.871736891	0.847498916
rs758049676	mosquito_bit_more	196102	160624	1.68E-08	0.02901153	0.005142788	0.851970758	0.86891276
rs758049676	multiple_sclerosis	10472	1646247	7.34E-10	-0.08964621	0.014591011	0.863582774	0.823635764
rs758049676	mycin_drug_allergy	19758	1050772	2.27E-06	-0.05060463	0.01071551	0.871336376	0.845359265
rs758049676	no_allergies	969101	744269	4.59E-69	0.040565658	0.00230998	0.852705271	0.871500716
rs758049676	other_animal_allergy	40280	1044775	2.74E-18	-0.06612738	0.007593255	0.871399573	0.838509436
rs758049676	other_food_allergy	61878	1038378	1.06E-08	-0.03539192	0.006190189	0.871386476	0.854321129
rs758049676	other_plant_allergy	61869	1038270	2.71E-16	-0.05044038	0.006168742	0.871455292	0.84501405
rs758049676	penicillin_allergy	87996	1031087	1.67E-32	-0.06265616	0.0052848	0.871716979	0.841374038
rs758049676	pollen_allergy	254868	983756	1.29E-41	-0.0452987	0.003354166	0.87174735	0.847835478
rs758049676	psoriasis	87077	1520979	2.93E-10	0.032461999	0.00514787	0.862590153	0.87956813
rs758049676	rhinitis	277227	953957	5.64E-46	-0.04642034	0.003263291	0.871911906	0.848259152
rs758049676	seasonal_allergies_broad	297070	971775	6.77E-43	-0.04369888	0.003184672	0.871729904	0.849012618
rs758049676	severe_acne	169557	1401733	5.32E-105	-0.08406171	0.003869606	0.867236659	0.82941535
rs758049676	shellfish_allergy	24923	1049182	4.36E-07	-0.04813247	0.009536901	0.871288491	0.849025037
rs758049676	steroid_meds	260865	336193	2.07E-06	-0.01878798	0.003958286	0.867272706	0.857153572
rs758049676	stretch_marks_any	296832	400580	2.46E-17	-0.03355917	0.003962828	0.868172502	0.852910016
rs758049676	sulfa_drug_allergy	55114	1039623	2.61E-25	-0.06846644	0.006596199	0.871582307	0.837536752
rs758049676	t1d	12103	1634497	6.65E-14	-0.10159002	0.013593398	0.863657024	0.813985837
rs758049676	tonsillectomy	223231	395019	2.91E-17	-0.03517711	0.004163966	0.86643624	0.852866089
rs758049676	tree_nuts_allergy	19641	1050517	9.84E-08	-0.05704398	0.010717101	0.871312102	0.845943852
rs758049676	trees_allergy	181014	1004556	4.18E-35	-0.04754794	0.003849218	0.871704867	0.846682452
rs758049676	weeds_allergy	192578	1001777	1.28E-41	-0.05058872	0.003746386	0.87174763	0.844611478
